# Light chain modulates heavy chain conformation to change protection profile of monoclonal antibodies against influenza A viruses

**DOI:** 10.1038/s41421-019-0086-x

**Published:** 2019-04-16

**Authors:** Haixia Xiao, Tianling Guo, Mi Yang, Jianxun Qi, Chaobin Huang, Yuanyuan Hong, Jinjin Gu, Xuefei Pang, William Jun Liu, Ruchao Peng, John McCauley, Yuhai Bi, Shihua Li, Junxia Feng, Hailiang Zhang, Xupei Zhang, Xishan Lu, Jinghua Yan, Liling Chen, Yi Shi, Weizhi Chen, George Fu Gao

**Affiliations:** 1 Laboraroty of Protein Engineering and Vaccines, Tianjin Institute of Industrial Biotechnology, Chinese Academy of Sciences (CAS), Tianjin, 300308 China; 2 CAS Centre for Influenza Research and Early-Warning (CASCIRE), Chinese Academy of Sciences (CAS), China Research Network, Beijing, 100101 China; 3 CAS Key Laboratory of Pathogenic Microbiology and Immunology, Institute of Microbiology, Chinese Academy of Sciences (CAS), Beijing, 100101 China; 4 GENEWIZ China, Beijing, 102206 China; 5 College of Biotechnology, Tianjin University of Science and Technology, Tianjin, 300457 China; 6 National Institute for Viral Disease Control and Prevention, Chinese Centre for Disease Control and Prevention, Beijing, 102206 China; 7 WHO Collaborating Centre for Reference and Research on Influenza, Crick Worldwide Influenza Centre, The Francis Crick Institute, 1 Midland Road, London, NW1 1AT UK; 8 CAS Key Laboratory of Microbial Physiological and Metabolic Engineering, Institute of Microbiology, Chinese Academy of Sciences (CAS), Beijing, 100101 China; 9 Suzhou Centre for Disease Control and Prevention, Suzhou, 215004 China

**Keywords:** Immunology, Structural biology

## Abstract

The isolation of human monoclonal antibodies with broadly neutralizing breadth can provide a promising countermeasure for influenza A viruses infection. Most broadly neutralizing antibodies against influenza A viruses bind to the conserved stem region or the receptor-binding cavity of hemagglutinin and the interaction is dominated by the heavy chain. The light chain, however, contributes few or no direct contacts to the antigen. Here we report an H3-clade neutralizing human monoclonal antibody, AF4H1K1, which recognizes the hemagglutinin glycoproteins of all group 2 influenza A viruses. This human monoclonal antibody has been obtained through the screening by pairing different heavy and light chains from an H7N9-infected patient based on the next-generation sequencing technology. Further structural studies revealed that light chains modulate the neutralizing spectrum by affecting the local conformation of heavy chains, instead of direct interaction with the antigen. These findings provide important clues to understand the molecular basis of light chains in antigen recognition and to explore the strategies in particular of the use of light chain modification to develop broadly protective monoclonal antibodies against influenza A viruses and other emerging viruses.

## Introduction

Influenza virus is a serious pathogen that threatens global public health and infects both human and animal populations. In the last century, four influenza pandemics have been recorded to cause many human deaths: H1N1 in 1918 and 2009, H2N2 in 1957, and H3N2 in 1968^1–7^. This 2017/18 winter, with severe seasonal influenza attacks all over the world, from China to the United Kingdom and the United States (http://www.who.int/influenza/surveillance_monitoring/updates/latest_update_GIP_surveillance/en/), reminds us again the threat of a potential influenza pandemic. Of note, it is the centenary of the1918-flu pandemic, the most devastating pandemic in the history. Therefore, the current concern is understandable. Although vaccines against influenza A and influenza B viruses (IAVs and IBVs) are available, the protection they provided is limited, owing to rapid antigenic variation in the envelope proteins (hemagglutinin, HA; neuraminidase, NA) of influenza virus strains. In addition, IAVs have a natural reservoir in wild birds and pigs, from which pandemic viruses with novel *HA* and *NA* genes can emerge^8^. There is no pre-existing immunity against such emergent viruses in the human population and the available vaccines cannot protect against such strains. Thus, universal vaccines and therapeutic antibodies are urgently needed to fight off those rapidly changed influenza viruses. However, we are still on the way of generating a successful universal vaccine^9–12^ and yet face the annual challenge of the seasonal flu or pandemic influenza by occasion.

To date, great efforts have also been made to develop broadly neutralizing monoclonal antibody (bnMAb) as a therapeutic candidate against IAVs. Many bnMAbs targeting HA, such as F10, S139/1, CR6261, FI6v3, CR8020, F045–092, C05, CT149, VIS410, MHAA4549A, 54.a.39, and MEDI8852, have been described^13–24^. These HA-recognizing bnMAbs can efficiently neutralize IAVs with different mechanisms either by blocking virus entry or through inhibiting the virus-cell fusion. The use of Palivizumab^25^, an MAb on the market for the prophylaxis of respiratory syncytial virus has reinforced the therapeutic use of passive MAbs against viral infections and thus has promoted the clinical trials of anti-influenza virus MAbs. In recent years, several bnMAbs, including TCN-032 (Theraclone-Sciences, Inc., NCT01719874), CR8020 (Crucell Holland BV., NCT01938352), MHAA4549A (Genentech, Inc., NCT01980966), VIS410 (Visterra, Inc., NCT02468115), CR6261 (National Institute of Allergy and Infectious Diseases, NCT02371668) and MEDI8852 (MedImmune LLC, NCT02603952) have been evaluated in clinical trials and have shown promising results to reduce the virus shedding.

How the bnMAbs can neutralize different IAV subtypes is an intriguing question for virologists. Previous studies have shown that the bnMAbs bind to the conserved stem region of HA protein or the receptor-binding cavity and the binding interaction is dominated by the heavy chain^13,15–17,19,23,26^. Of note, one recent study showed that in vitro affinity maturation in both the heavy chain and light chain can engender a super bnMAb against all the IAV subtypes^24^. As an indispensable component for the MAb, the light chain has been largely neglected when considering the contribution to the bnMAb activity. Previous studies showed that the light chain can contribute to the binding and neutralization profile of human MAbs (huMAbs)^23,27,28^, but the molecular mechanism is yet unknown, providing little information for the engineering of more powerful bnMAb targeting the light chain.

In the current study, we used next-generation sequencing (NGS) technology to establish a pool of heavy chain and light chain variable region (V) genes from an H7N9-infected patient, and obtained a series of huMAbs by pairing the most frequently occurring V genes of heavy and light chains. We found that the huMAbs generated from a certain heavy chain paring with different light chains can have different binding abilities to the HA proteins, from no binding to broad binding. Fortunately, we obtained one neutralizing MAb against IAVs, named as AF4H1K1, which can effectively neutralize H3-clade viruses in vitro and possess the prophylactic and therapeutic efficacy against both H3 and H7 subtype IAVs in vivo. Further structural analysis demonstrates that the epitope recognized by AF4H1K1 is located in the HA stem region and the light chain K1 can modulate local conformation of paired heavy chain to change the protection profile, compared with the non-binding AF4H1K1 single chain variable fragment (scFv) and AF4H1L1 Fab molecules.

## Results

### Antibody screening by NGS technology

A pool of variable region genes of B-cell receptors (including λ-light chain, κ-light chain and heavy chain) from an H7N9-infected patient’s peripheral blood mononuclear cells (PBMCs) were amplified, sequenced, and analyzed with NGS. According to the abundance and length of the complementarity-determining region 3 (CDR3) sequence (Supplementary Table S1), 3 heavy chain V genes, and 5 light chain V genes (containing 3 κ-chains and 2 λ-chains) were selected (Table 1), and 15 paired IgG antibodies were generated. Among those paired IgGs, we found that only one paired IgG with heavy chain H1 and light chain K1 could neutralize H3N2 IAV rather than H7N9 in vitro (Supplementary Table S2). This MAb was named as AF4H1K1. Of note, other expressed H1-paired IgGs did not show the ability to neutralize H3N2 or H7N9 viruses (Supplementary Table S2).

**Table 1:** Isotype and CDR3 sequence analysis for the selected three heavy chains and five light chains from an H7N9-infected patient

Heavy/light chain	V-gene and allele	D-gene and allele	J-gene and allele	CDR3 sequence	SHM nt (aa)	Frequency (%)
H1	IGHV3–30–3*01	IGHD3–16*01	IGHJ4*03	ARDPLTKLLPFDWVSGGYFDY	19 (13)	2.8
H2	IGHV2–5*02	IGHD6–19*01	IGHJ4*01	AHFGQWLVYFDY	7 (5)	2.6
H3	IGHV3–30*01	IGHD3–10*02	IGHJ6*03	ARDVYDFWSEPFNYDFCYMDV	10 (7)	1.9
K1	IGKV3–20*01	-	IGKJ1*01	QQYGSSFT	5 (3)	12.2
K2	IGKV2–30*01	-	IGKJ1*01 or IGKJ4*01	MQGTHWPLS	9 (5)	2.5
K3	IGKV1–39*01 or IGKV1D-39*01	-	IGKJ1*01	QQSYSTPYT	7 (3)	2.3
L1	IGLV3–1*01	-	IGLJ1*01	QAWDSSTVV	0 (0)	23.0
L2	IGLV2–14*01	-	IGLJ1*01	SSYTSSSTLV	2 (1)	4.4

*aa* amino acid, *nt* nucleotide, *SHM* somatic hypermutation

### AF4H1K1 could neutralize H3-clade IAVs

Having obtained a huMAb AF4H1K1, which could neutralize H3N2 IAV, we then tested its neutralization activities to other subtypes of IAVs in vitro. The results showed that AF4H1K1 could neutralize H3-clade IAVs, including H3, H4, and H14 with 50% inhibitor concentration (IC_50_) arranging from 13.2 μg/ml to 189.0 μg/ml (Table 2). AF4H1K1 could recognize all subtypes of HA proteins expressed in group 2 IAV-infected cells’ surface (Supplementary Fig. S1a, b), but not HA proteins presented in group 1 IAV-infected cells (Supplementary Fig. S1c). AF4H1K1 binds most of group 2 HAs with high affinities, with *K*_D_ values from 4 pM to 49 nM for H3, H4, H14, H7, and H10 HAs, respectively (Supplementary Table S3). By contrast, AF4H1K1 has a much lower affinity to the H15 HA, with a *K*_D_ value of ~1.8 μM (Supplementary Table S3).

**Table 2:** Neutralization activity of AF4H1L1, AF4H1K1, and AF4H1/FI6L against a panel of IAVs from different groups

Groups		Subtypes	Virus strains	Abbreviations	Neutralization titer with different MAbs (IC_50_, μg/ml)		
					AF4H1L1	AF4H1K1	AF4H1/FI6v3L
Group 2	H3-clade	H3N1	H3_(A/Aichi/2/1968)_/PR8-RG	H3-rAC/68	> 200^a^	138.0	ND
		H3N2	A/Jiangxi//262/2005	H3-JX/05	> 200	26.5	23.2
		H4N1	H4_(A/duck/Czech/1956)_/PR8-RG	H4-rCZ/56	> 200	189.0	150.0
		H14N5	A/mallard duck/Astrakhan/263/1982	H14-AS/82	> 200	13.2	16.7
	H7-clade	H7N9	A/Anhui/1/2013	H7-AH/13	> 200	> 200	71.1
		H10N8	A/Jiangxi-Donghu/346/2013	H10-JX/13	> 200	> 200	66.9
		H15N8	A/duck/Australia/341/1983	H15/AU/83	> 200	> 200	> 200
Group 1		H1N1	A/California/04/2009	H1-CA/09	> 200	> 200	> 200
		H2N3	A/environment/Guangdong/2/2009	H2-GD/09	> 200	> 200	> 200
		H5N1	A/bar-headed goose/Qinghai/1/2005	H5-QH/05	> 200	> 200	> 200
		H9N2	A/chicken/Beijing/2/1997	H9-BJ/97	> 200	> 200	> 200

^a^The starting concentration of MAbs for the neutralization is 400 μg/ml. *ND* not done

### Light chain contributes to the neutralizing activity and spectrum

Heavy chain variable region (VH) of AF4H1K1 shares high identity with VH of FI6v3, a pan-IAV-neutralizing huMAb^16^. We have shown that pairing of different light chains can change the binding capacity and neutralizing activity, and then want to see what will happen to the neutralizing breadth of the AF4H1/FI6v3L, a hybrid MAb with heavy chain from AF4H1K1 and light chain from FI6v3. To this end, we prepared the hybrid MAb AF4H1/FI6v3L and tested its neutralizing activity against different subtypes of IAVs in parallel with AF4H1L1 and AF4H1K1. The data clearly showed that AF4H1L1 has no neutralizing activity with either group 1 or group 2 IAVs (Table 2). The neutralizing breadth of AF4H1K1 was restricted to H3-clade IAVs in vitro, including H3, H4, and H14 subtypes (Table 2). Interestingly, AF4H1/FI6v3L expands the neutralizing breadth to both H3- and H7-clade IAVs in vitro (Table 2). AF4H1/FI6v3L has lower neutralizing activity toward H7 and H10 IAVs, with IC_50_ values of 71.1 μg/ml and 66.9 μg/ml, respectively. However, this hybrid MAb failed to expand its neutralizing activity to group 1 IAVs (Table 2).

### Crystal structures of AF4H1K1/H3 and AF4H1K1/H4 complexes

**Fig. 1:**
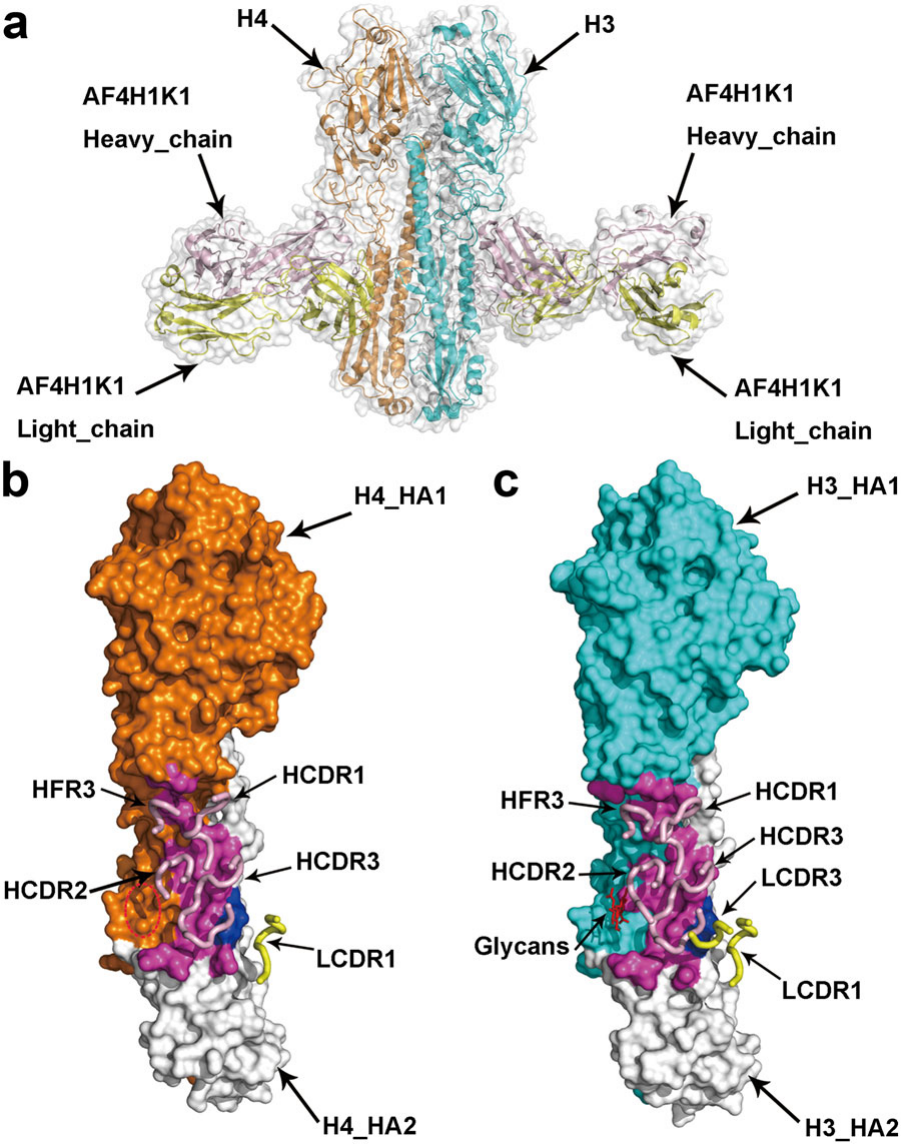
AF4H1K1 binding to the fusion subdomain of the HA trimer. **a** Trimer of H3/H4 HA-binding AF4H1K1. The structures of HA and Fab are in ribbons representation. The figure was generated by superimposing the structures of AF4H1K1 Fab in complex with either H3 or H4 trimer. Only one HA monomer bound to one AF4H1K1 Fab is shown for each complex for comparison. H3 HA is in cyan, H4 HA is in orange, AF4H1K1 heavy chain is in light pink, and light chain is in yellow. The individual HA-Fab complex structures are shown in Supplementary Fig. S2. The binding footprint of AF4H1K1 in the H4 HA (**b**) and H3 HA (**c**) is in surface representation. The epitope of H4 or H3 recognized by AF4H1K1 VH is presented with purple, whereas the amino acids interacted with VL is labeled with blue. HA2 subunits of H4 HA and H3 HA are in white color. The glycan molecules on the Asn38 of H3 HA1 are in red and the corresponding position of 38 at H4 HA1 is circled with red-circled dashed line

**Fig. 2:**
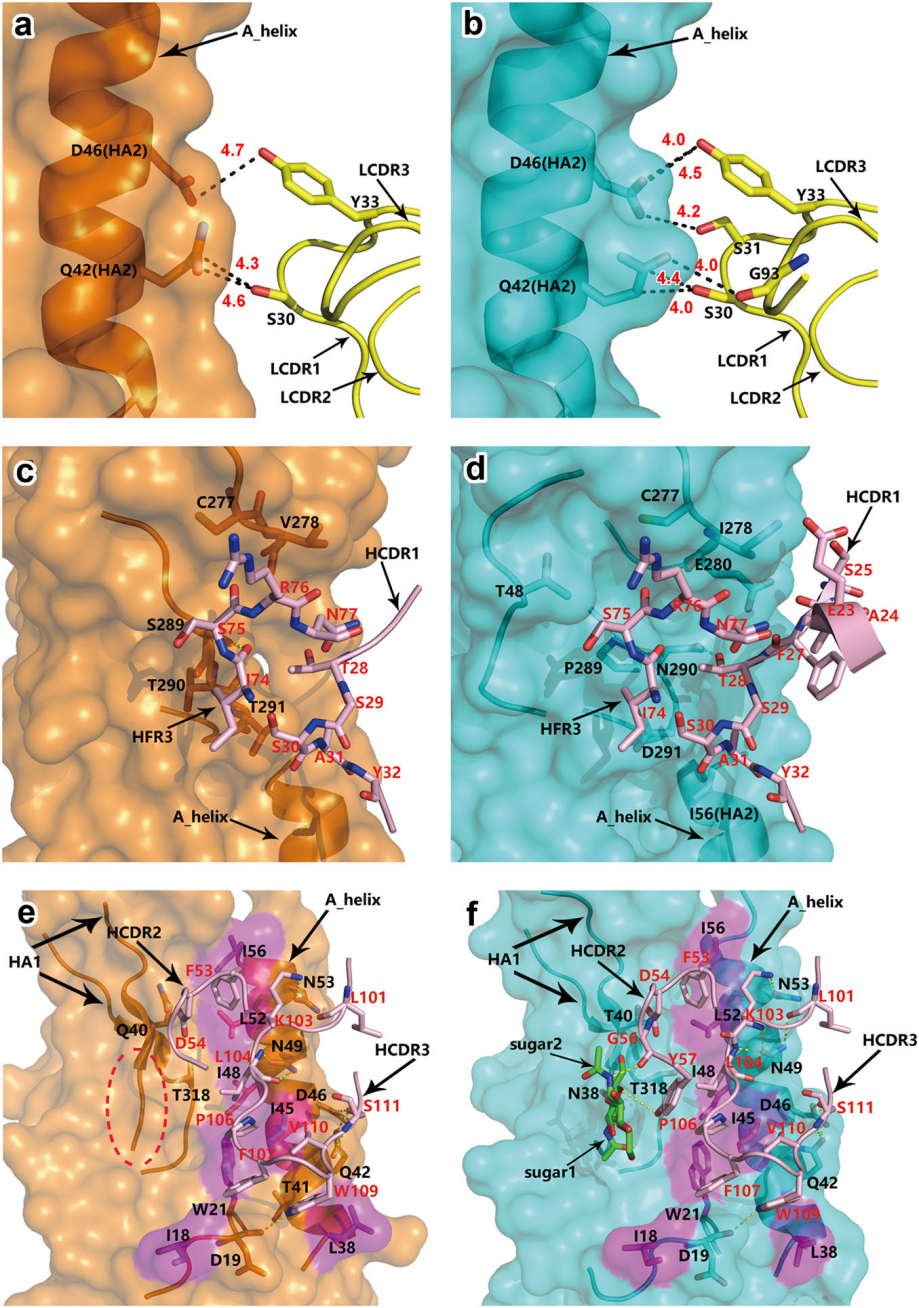
The interaction of AF4H1K1 VL and VH with H3 HA and H4 HA. Interactions of AF4H1K1 VL with H4 HA2 (**a**) and H3 HA2 (**b**) are presented, respectively. The labels of amino acids in the HA and AF4H1K1 VL are in black color, whereas the others in the AF4H1K1 VH are in red. AF4H1K1 VL (yellow) makes weak interactions by van der waals contacts (black dashed line) with Gln42 and Asp46 in the HA2 of H4 HA (**a**) and H3 HA (**b**). The distance of each contact was labeled in red number. The upper region of the binding interface between AF4H1K1 and HA. Amino acid residues in the HFR3 and HCDR1 of AF4H1K1 VH (light pink) recognize HA1 vestigial-esterase domains mediated by van der Waals contacts and a single hydrogen bond in both AF4H1K1/H4 (**c**) and AF4H1K1/H3 (**d**) structures. The lower region of the binding interface between AF4H1K1 and HA. HCDR2 and HCDR3 loops insert into the cleft between the two helices of HA2 and mainly contact with the A-helix by hydrophobic interactions and hydrogen bonds (yellow dashed lines) in both AF4H1K1/H4 (**e**) and AF4H1K1/H3 (**f**) structures. In addition, glycans on the Asn38 of HA1 contribute two hydrogen bonds to the interaction (**f**). Red-circled dashed line indicates the absence of a glycan in the H4 HA (**e**)

To define the epitopes recognized by AF4H1K1, we determined the crystal structures of the AF4H1K1 Fab in complex with HAs from both the 1968 H3N2 pandemic isolate (H3-AC/68) and the H4N6 duck isolate (H4-CZ/56) at 2.9 Å and 3.8 Å resolutions, respectively (Supplementary Table S4). The overall structures of the two complexes are very similar. The AF4H1K1 Fab fragment binds both H3 HA and H4 HA at the stem and vestigial-esterase domains with a similar orientation, where the Fab molecule is roughly orthogonal to the HA stalk (Fig. 1a-c; Supplementary Fig. S2a, b). In both complex structures, the heavy chain dominates the interactions with HA molecules (Fig. 1b, c). The binding footprint of AF4H1K1 heavy chain involves both HA1 and HA2 subunits, whereas the light chain contacts with only two amino acids, Gln42 and Asp46 of HA2 subunit, which are also involved in heavy-chain interactions (Fig. 2a, b, e, f). In AF4H1K1 heavy chain, the heavy chain CDR1 (HCDR1), HCDR2, HCDR3, and heavy chain framework region 3 (HFR3) loops participate in the interactions to both H3 and H4 HA molecules (Fig. 1b, c and Fig. 2c-f). In comparison, only the Ser30, Ser31, and Tyr33 of light chain CDR1 (LCDR1) and Gly93 of LCDR3 contribute to H3 HA epitope recognition, whereas the LCDR3 loop is not involved in recognizing H4 molecule (Fig. 1b, c, 2a, b).

The binding interface of AF4H1K1 heavy chain is divided into upper and lower regions, which involve mainly HA1 vestigial-esterase domain and HA2 fusion domain, respectively. In the upper region, the HCDR1 and HFR3 loops protrude into the pit formed by the vestigial-esterase domain and HA2 stalk connecting region (Fig. 1b, c). The interactions in this region are mainly mediated by van der Waals’ contacts combined with a single hydrogen bond in both structures. In AF4H1K1/H3 complex the hydrogen bond is formed between the side chains of Thr28 in HCDR1 and Asn290 in HA1 (Fig. 2d), whereas for AF4H1K1/H4 complex the hydrogen bond connects the carbonyl group of Ile74 in HFR3 loop and the side chain of Ser289 in HA1 (Fig. 2c).

The HA/Fab interactions are dominated by the lower interface in HA2 fusion domain. In this region, the HCDR2 and HCDR3 loops of AF4H1K1 insert into the cleft between the two helices of HA2 domain and mainly contact with the A-helix (Fig. 1b, c and Fig. 2e, f). The interactions mainly involve hydrophobic interactions and hydrogen bonds. Four non-polar amino acids at the base of HA2, Ile18, Trp21, Leu38, and Ile45, form an open hydrophobic pocket to accommodate the bulky aromatic side chains of Phe107 and Trp109 in HCDR3 loop (Fig. 2e, f). Along the A-helix, Ile48, Leu52, and Ile56 extend the hydrophobic surface to interact with Leu104 and Pro106 in HCDR3 and Phe53 in HCDR2 (Fig. 2e, f). These amino acids together form a continuous hydrophobic interface along the HA2 stalk, which mainly contributes to epitope recognition. In addition, four polar amino acids at the outer surface of A-helix, Gln42, Asp46, Asn49, and Asn53 form hydrogen bond networks with the main chain of Leu101, Lys103, Leu104, and Ser111 in the HCDR3 loop (Fig. 2e, f). The charged side chain of Lys103 also participates in the interaction with Asn53 of HA2 by strong hydrogen binding (Fig. 2e, f). These three amino acids, Gln42, Asp46 and Asn53, in A-helix are highly conserved among all HA subtypes (Supplementary Fig. S3).

Despite the high similarity between the binding profiles for H3 HA and H4 HA, the AF4H1K1 heavy chain still harbors a notable difference in the recognition of the two HA subtypes. In AF4H1K1/H3 complex, there is an *N*-linked glycosylation modification at Asn38 of HA1, where the glycan directly interacts with Gly56 and Tyr57 of HCDR2 loop by hydrogen bonds (Fig. 2f). However, this glycosylation site is absent in H4 HA and thus the HCDR2 loop is less competent for recognizing H4 HA as compared with H3 HA (Fig. 1b, c and Fig. 2e, f).

### Structure of AF4H1L1 Fab and AF4H1K1 scFv molecules

To explore the reason why only the combination of heavy chain H1 and light chain K1 rather than other H1-paired IgGs with K3, L1, or L2 light chains from the H7N9-infected patient is capable of binding group 2 HAs and neutralizing H3-clade IAVs, we determined the crystal structure of non-binding AF4H1L1 Fab at 2.1 Å resolution. AF4H1L1 shares the same heavy chain H1 with AF4H1K1 but contains a different light chain L1. AF4H1L1 could not bind HAs from group 2, such as H3, H4, H14, H7, H10, and H15, as shown by surface plasmon resonance (SPR) assay (Supplementary Table S3). In consistence with the binding test, AF4H1L1 could not neutralize IAVs from either group 1 or group 2 (Table 2).

**Fig. 3:**
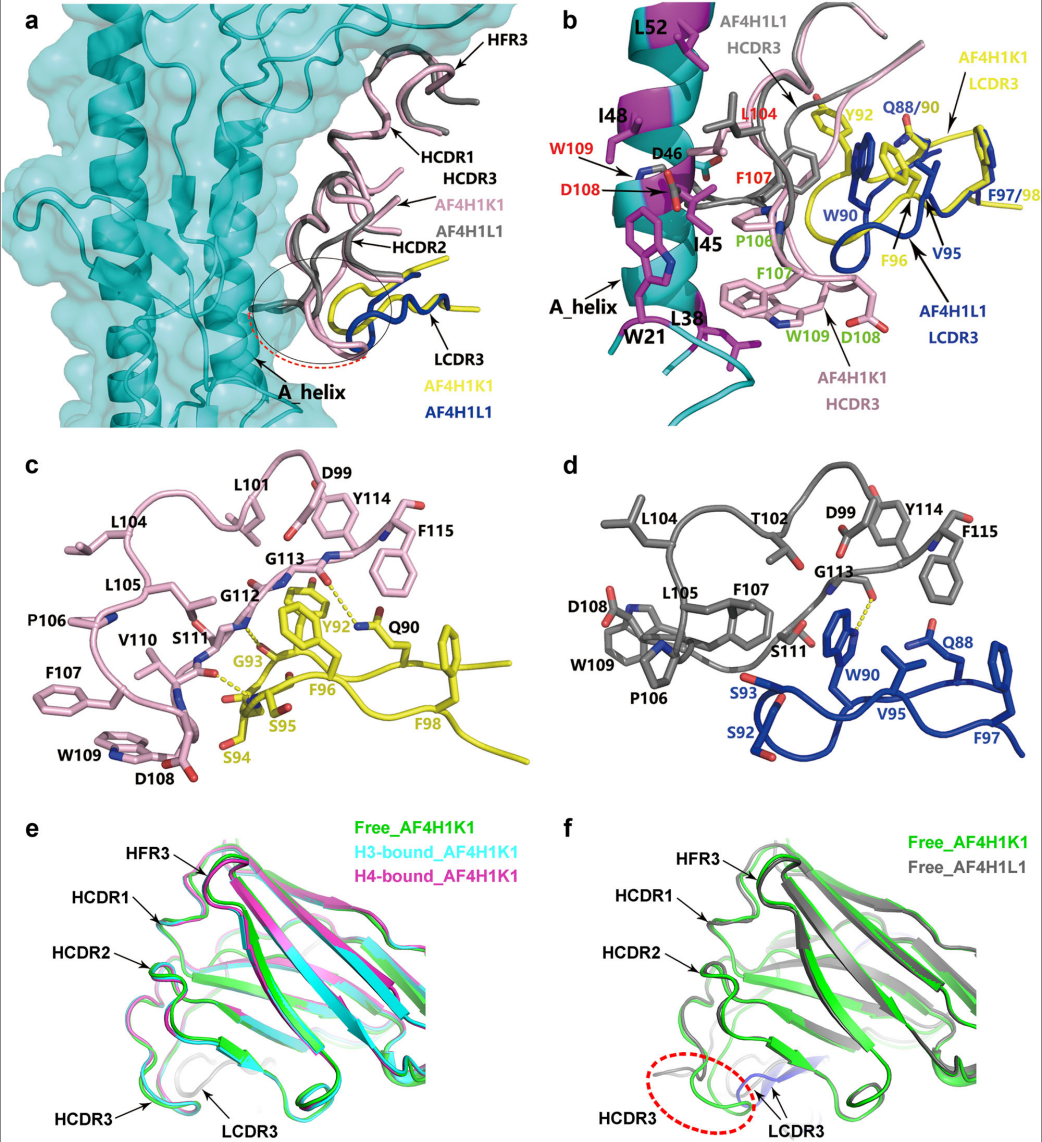
Structural comparison between AF4H1K1 and AF4H1L1. **a** AF4H1L1 Fab is overlapped with AF4H1K1/H3 complex. H3 HA is labeled with cyan, AF4H1K1 Fab VH in complex with H3 HA is in light pink, and the VL is in yellow. AF4H1L1 VH is in gray color and VL is in marine. Circled area shows the conformation difference between AF4H1K1 and AF4H1L1, and the red dashed arrow line suggests the flipping over of AF4H1L1 HCDR3 toward A-helix comparing AF4H1K1 HCDR3. **b** VH and VL CDR3 loops from both AF4H1K1 and AF4H1L1 Fab fragments are highlighted. The labels of amino acids in the HA are in black color, the residues in the AF4H1K1 HCDR3 are in green, those in AF4H1L1 HCDR3 are in red, those in AF4H1K1 LCDR3 are in yellow, and the others in AF4H1L1 are in marine. It is clearly that HCDR3 of AF4H1L1 flippers over toward the HA A-helix and causes fierce clash between Asp108, Trp109 in HCDR3 and Ile45, Asp46 in the A-helix. **c** Hydrogen bonds between LCDR3 and HCDR3 of AF4H1K1. LCDR3 makes contacts with HCDR3 mainly by hydrogen bonds (yellow dashed line) between Ser111, Gly112, Gly113 of HCDR3 and Gln90, Gly93, Ser94, or Ser95 of LCDR3. **d** Contacts between HCDR3 and LCDR3 of AF4H1L1. HCDR3 and LCDR3 of AF4H1L1 mainly involved hydrophobic interactions and one hydrogen bond. Trp90, Val95 in LCDR3, and Leu105, Pro106, Phe107, Trp109, and Phe115 in HCDR3 are the amino residues for the hydrophobic interaction. **e** Unbound AF4H1K1 scFv molecule (green) is overlapped with Fab molecules from H3-bound (cyran) and H4-bound (magenta) AF4H1K1. All the CDR loops adopt similar conformations in both apo and HA-bound states. **f** Unbound AF4H1K1 scFv molecule (green) is overlapped with AF4H1L1 Fab molecules (gray). The different conformations of HCDR3 are highlighted by a red dashed oval

To figure out the structural basis for its inability to bind group 2 HAs, the AF4H1L1 Fab structure was super-imposed onto the AF4H1K1 Fab in AF4H1K1/H3 complex. As shown in the pseudo-complex structure of AF4H1L1/H3, the heavy chain adopts a similar conformation to interact with the HA molecule and has a dominant role in epitope recognition as the case of AF4H1K1 (Fig. 3a). The HCDR1, HCDR2, and HFR3 loops all super-impose well between the two Fab structures. However, significant conformation change was observed at the HCDR3 loop when paired with different light chains (Fig. 3a).

In AF4H1K1 Fab, the HCDR3 loop bends backward to avoid the A-helix of HA2, allowing enough space for the bulky side chains of Phe107 and Trp109 in the hydrophobic pocket at the base of A-helix (Fig. 3b). In contrast, the HCDR3 loop of AF4H1L1 Fab directly protrudes toward the A-helix without bending, thus causes severe clash between Asp108, Trp109 in HCDR3 and Ile45, Asp46 in the A-helix (Fig. 3a, b). This conformation change of HCDR3 was induced by the different light-chain interaction modes in AF4H1L1 Fab. In AF4H1K1 Fab, the amino acids Gln90, Gly93, and Ser94 in LCDR3 loop form a continuous hydrogen bond pattern with the main chain of Ser111, Gly112, and Gly113 in HCDR3, which drags the HCDR3 loop to fold back so that to hinder the neighboring Trp109 and Phe107 side chains from flipping upward (Fig. 3c), whereas in AF4H1L1 Fab the hydrogen bond network between the main chains of HCDR3 and LCDR3 is interrupted to accommodate the bulky side chain of Trp90 in LCDR3. Instead, the Trp90 and Val95 in LCDR3 form a hydrophobic core (Fig. 3d). As the restrains of the hydrogen bond network is lost, the HCDR3 is released and the side chain of Phe107 flaps upward and bends toward Trp90 of LCDR3 due to hydrophobic interactions; thus, the neighboring Asp108 and Trp109 together move upward and collide with the A-helix (Fig. 3b, d). This change makes it impossible for AFH1L1 Fab to bind HA stem, thus abolishing its neutralizing activity.

To further confirm that the different conformations between HCDR3 from H3-bound or H4-bound AF4H1K1 and HCDR3 from AF4H1L1 are due to the allosteric effect of the paired light chain, but not due to that induced by the HA binding, we solved the crystal structure of free AF4H1K1 scFv at 1.4 Å resolution as well. Of note, HCDR1, HCDR2, HCDR3, and HFR3 of the unbound AF4H1K1 scFv possess the same conformation with the ones from the H3-bound and H4-bound AF4H1K1 Fab molecules (Fig. 3e). Compared with the free AF4H1K1 scFv molecule, the HCDR3 of AF4H1L1 does flip away from the LCDR3 (Fig. 3f). This demonstrates that the conformational change of AF4H1L1 is driven by the paired light chain but not the result of induced fit due to HA binding.

To determine the contribution of K1 CDR3 to the binding and neutralization activity, we produced one mutant IgG, AF4H1/L1CDR3m, which replaced the L1 CDR3 residues with those of K1 (Supplementary Fig. S4c). The results clearly showed that AF4H1/L1CDR3m neither could bind to H3 HA (Supplementary Fig. S5a) or H7HA protein (Supplementary Fig. S5b), nor presented any neutralizing activity to H3 and H7 IAVs (Supplementary Table S2). These results suggested the conformation of K1 CDR3 is correlated with other neighboring residues in the light chain and simple introduction of K1 CDR3 in L1 cannot result in the same CDR3 conformation as in K1.

### Structural comparison between AF4H1K1 and FI6v3 Fabs

Based on sequence analysis, we found that VH of AF4H1K1 is from IGHV3–30–3*01 allele and it shares a high degree of sequence identity with the VH from FI6v3 HuMAb^16^. The differences between AF4H1K1 VH and FI6v3 VH mainly locate at the HFR3, HCDR1, HCDR2, and HCDR3 regions (Supplementary Fig. S4a). AF4H1K1 light chain variable region (VL) is quite different from that of FI6v3 (Supplementary Fig. S4b); the former is from IGKV3–20*01 allele (Table 1), and the latter is from IGKV4–01*01^16^.

**Fig. 4:**
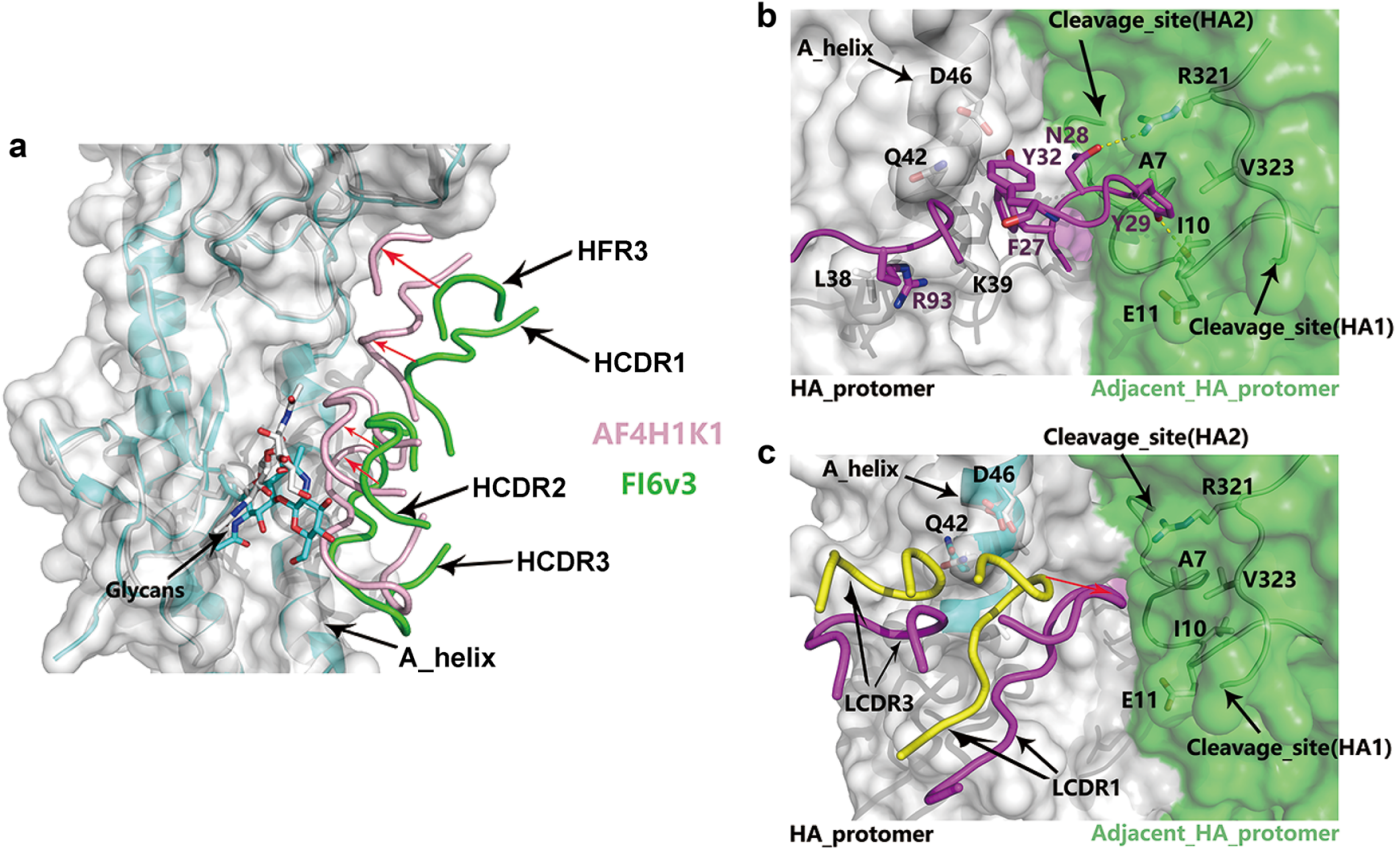
Comparison of HA fusion subdomain-interacted domains of VH and VL from AF4H1K1 and FI6v3. **a** Structural overlapping between AF4H1K1/H3 complex and FI6v3/H3 complex (PDB ID, 3ZTJ). AF4H1K1 VH is in light pink and the FI6v3 VH is labeled with green. H3 HA binding to AF4H1K1 is present in cyan and H3, which is in complex with FI6v3, is in white. Red arrows suggest the lift up of AF4H1K1 key components toward HA compared with FI6v3. **b** Interaction of FI6v3 VL with two H3 HA protomers (PDB ID, 3ZTJ). The structure is in surface presentation, with one protomer in white color and the other in green. FI6v3 LCDR3 (purple) reaches out to the fusion peptide of the neighboring HA protomer and forms two hydrogen bonds between LCDR3 Asn28, Tyr29 and neighboring HA1 Arg321, HA2 Ile10. Labels for FI6v3 LCDR are in purple and the others of HA are in black. **c** Structural overlapping between AF4H1K1/H3 complex and FI6v3/H3 complex (3ZTJ) in the light chain–HA interface. AF4H1K1 light chain LCDR1 loop (yellow) interacts with two amino acid residues at HA2 stalk (cyan): one is Gln42 and the other is Asp46. There is no direct interaction between AF4H1K1 light-chain LCDR3 loop and HA (cyan), whereas both LCDR1 and LCDR3 loops of FI6v3 light chain are involved in the interaction with HA fusion subdomain (white). FI6v3 LCDR1 shows weak attachment to HA2. In addition, FI6v3 LCDR3 reaches out to the fusion peptide of the neighboring HA in FI6v3/H3 complex

To illustrate the differences in the epitope recognition by AF4H1K1 and FI6v3, we compared the complex structures of FI6v3/H3 and AF4H1K1/H3 by superimposing the two H3 HA molecules together. Compared with FI6v3, the AF4H1K1 VH, including HFR3, HCDR1, HCDR2, and HCDR3, makes much closer contacts with H3 HA (Fig. 4a). The HCDR loops and HFR3 loop of AF4H1K1 are universally lifted up and protrude closer to the H3 HA molecule, especially the HFR3 and HCDR1 loops. Thus, these loops contribute more in epitope recognition as compared with FI6v3. In addition, the closer contacts between AF4H1K1 HCDR2 and HCDR3 with HA fusion subdomain also enhance their interactions with A-helix, as well as the glycans linked to Asn38 of HA1 (Fig. 4a).

FI6v3 is a broadly neutralizing antibody with reactivity against nearly all subtypes of IAVs, whereas AF4H1K1, which shares a high sequence identity with FI6v3 VH, could only neutralize the H3-clade viruses in vitro. Moreover, the H1-paired IgGs with light chain K3, L1, or L2 were unable to neutralize any subtype in vitro. All these results indicate the importance of light chains in modulating the neutralizing spectrum of MAbs. We performed sequence alignment for variable regions of AF4H1K1 and FI6v3 light chains and observed that LCDR1, LCDR2, and LCDR3 regions are highly variable among these light chains (Supplementary Fig. S4b). Notably, FI6v3 possesses a longer LCDR1 loop, which facilitates the FI6v3 light chain to interact with the fusion peptide of another adjacent HA protomer (Fig. 4b) rather than altering the HCDR3 conformation. In the LCDR1 loop of FI6v3, side chains of Asn28 and Tyr29 form two hydrogen bonds with Arg321 and Glu11 of the neighboring HA, which results in the pan-IAV-neutralizing activity. In contrast, the AF4H1K1 light chain, with a shorter LCDR1 loop, only interacts with the A-helix of HA2 by a few weak van der Waals’ contacts, which could also restrain its neutralizing spectrum (Fig. 2a, b, 4c). Therefore, the longer LCDR1 loop from FI6v3 light chain probably contributes to expand the neutralizing breadth of AF4H1/FI6v3L MAb by directly contacting HA.

### Mechanism of neutralization

AF4H1K1 prevented syncytia formation in H3N2-infected BHK21 cells when the pH of the medium dropped to 5.0 (Supplementary Fig. S6a) and the negative control IgG could not (Supplementary Fig. S6b). To test whether the binding of AF4H1K1 to HA0 could block the immature H3-clade HA0 cleavage and hence inhibit the H3-clade viruses’ infectivity, we incubated AF4H1K1 with different HA0 proteins including H3, H4, H14, and H15 in different molar ratios. The AF4H1K1-HA0 mixture was exposed to tosyl phenylalanyl chloromethyl ketone (TPCK)-treated trypsin for 15 min (min) at 37 °C and then was loaded on SDS-polyacrylamide gel electrophoresis (PAGE). The trypsin digestion results suggested that AF4H1K1 could inhibit the cleavage of H3, H4, and H14 HA0s by trypsin with the increase of MAb concentration (Supplementary Fig. S7a). Furthermore, pretreatment of mature H3-clade HAs with AF4H1K1 blocked the pH-induced conformational change, retaining HAs of H3, H4, and H14 in the protease-resistant, prefusion state (Supplementary Fig. S7b). On the contrary, pretreatment of H15 with AF4H1K1 at a ratio of 3:1 did not inhibit the HA0 cleavage or block the conformational change, which may be the result of the low affinity (*K*_D_ value is 1.8 μM). Based on these in vitro data (Supplementary Fig. S6, S7) and the AF4H1K1/H3 or AF3H1K1/H4 complex structure, we propose that the mechanism of AF4H1K1 neutralization is the result of blocking the membrane fusion between virus and cell membrane.

### Prophylactic and therapeutic efficacy of AF4H1K1

**Fig. 5:**
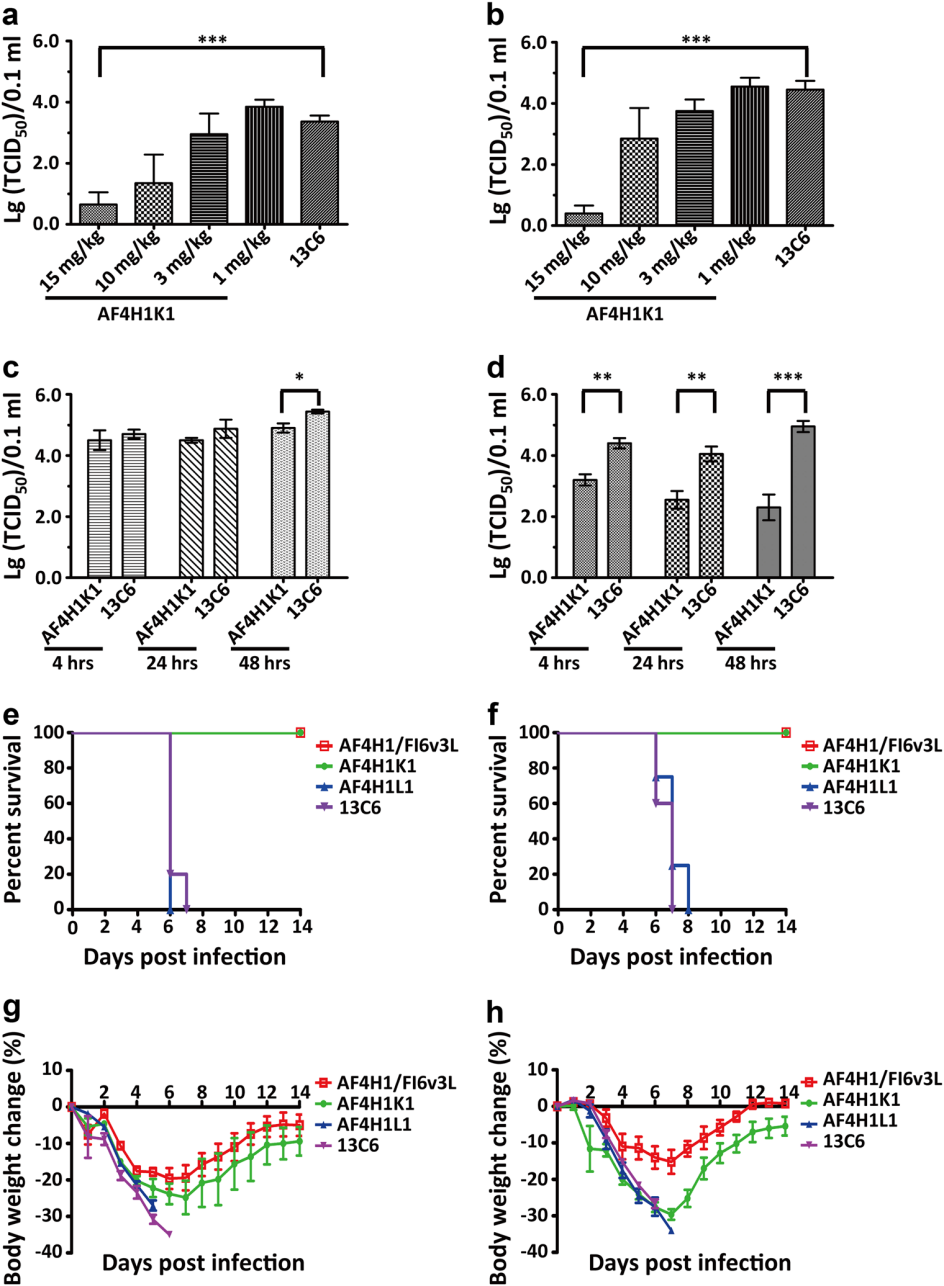
AF4H1K1 showed prophylactic and therapeutic activities against H3 and H7 IAVs. **a**–**d** Prophylactic and therapeutic efficacies of AF4H1K1 against H3-rAC/68. In the H3-infected experiments, BALB/c mice were intravenously administrated with 15 mg/kg of AF4H1K1 via tail veil 1 day before, or 4, 24, and 48 hours (hrs) after being challenged with 100 MID_50_ (15 mice per group). Anti-Ebola MAb 13C6-treated mice were used as a control. Lung tissues were collected, homogenized for the virus titration at 3 dpi (**a**, **c**) and 5 dpi (**b**, **d**), respectively. AF4H1K1 could reduce the virus replication in the lung of virus-infected mice in both preexposure prophylactic evaluation (**a**, **b**) and therapeutic assessment tests (**c**, **d**) according to the unpaired *t*-tests (*n* = 5) using GraphPad Prism 5 software. *P < 0.05, **P < 0.01, ***P < 0.001. They are one representative experiment out of three. **e**–**h** Evaluation of prophylactic and therapeutic efficacies of AF4H1K1 against H7N9 HPAIV. In the H7N9 IAV-challenged experiments, BALB/c mice were intravenously administrated with 15 mg/kg of AF4H1K1 via tail veil at 1 day  before or after virus challenge with 10^5^ 50% of lethal dose (LD_50_) (equally to 10^7^ 50% egg infective dose (EID_50_)). AF4H1K1 and AF4H1/FI6v3L were fully protective against the lethality when being administrated at 24 h before (**e**) and after (**f**) virus challenge, but could not prevent the body weight from dropping in both prophylactic (**g**) and therapeutic (**h**) experiments. *n* = 5. They are one representative experiment out of two, which have been done in Biosafety level 3 laboratory. Survival analysis was carried out by Kaplan–Meier method using GraphPad Prism 5 software

To evaluate the protective efficacy of AF4H1K1 IgG against H3 subtype IAV in vivo, we rescued one recombinant virus H3-rAC/68 (*HA* from H3N2 A/Aichi/2/1968 and the remaining seven gene segments from H1N1 A/Puerto Rico/8/1934). This recombinant virus, H3-rAC/68, does not cause death in the infected mice but it replicates well in the lung of virus-infected animals. Therefore, we used the viral titer in the lung as an index to determine the protective efficacy of AF4H1K1 and determined the 50% mouse infectious dose (MID_50_) based on the viral replication in the lung. For the prophylactic test, virus titers in the lungs of mice treated with 15 mg/kg AF4H1K1 1 day before challenge were reduced by around 500-fold and 10,000-fold at 3 and 5 days post infection (dpi), respectively. In contrast, the non-reacting anti-Ebola virus MAb 13C6 did not confer any obvious effect on virus titer, which was used as a negative control (Fig. 5a, b). Moreover, AF4H1K1 also showed the ability to inhibit H3-rAC/68 replication in the therapeutic evaluation test (Fig. 5c, d). After 4, 24, and 48 h post virus challenge, H3-rAC/68-infected mice were treated with AF4H1K1 or 13C6 IgG. On 3 dpi, virus titer in the lung of AF4H1K1-treated group at 48 h was lower than that of control group (Fig. 5c), whereas on 5 dpi all three AF4H1K1-treated groups at different time points (4, 24, and 48 h) significantly inhibited the virus replication comparing with the 13C6-treated group (Fig. 5d). These data indicate that AF4H1K1 has both prophylactic and therapeutic efficacy against H3-rAC/68 infection in vivo.

As previous work showed that CR9114, one IBVs-binding bnMAb, did not show in vitro neutralizing activity against IBVs, but could provide full protection from lethal challenge of IBVs^26^, we evaluated the prophylactic and therapeutic efficacy of AF4H1K1 against the lethal challenge in mice with a newly isolated H7N9 strain, which has multiple basic amino acids at the HA cleavage site^29^. Most of the key residues in H3 and H4 recognized by the AF4H1K1 are highly conserved between the 2013 low pathogenic avian influenza virus H7N9 and 2017 highly pathogenic avian influenza virus (HPAIV) H7N9 (Supplementary Fig. S8). The administration of AF4H1K1 and AF4H1/FI6v3L at 15 mg MAbs per kg of body weight at 1 day before or after virus challenge could fully protect the mice from the lethal challenge by a high dose (10^5^ 50% lethal dose) of H7N9 HPAIV (Fig. 5e, f), although weight loss was not completely prevented (Fig. 5g, h). However, all the mice administrated with 13C6 or AF4H1L1 at both prophylactic and therapeutic evaluation groups died at 7 or 8 dpi (Fig. 5e, f). These data revealed that AF4H1K1 has both prophylactic and therapeutic efficacy against H7N9 IAV lethal challenge in vivo.

## Discussion

To isolate neutralizing HuMAb for H7N9 IAV, we initiated this study with PBMCs sample from an H7N9-infected patient using NGS technology. At the beginning of this study, we selected three VH and five VL candidates to make 15 pairs of IgG. It turned out that only one paired IgG with H1 and K1 can neutralize IAVs. This HuMAb, designated AF4H1K1, is able to neutralize H3-clade viruses (H3, H4, and H14), rather than H7-clade viruses (H7, H10, and H15) of group 2 viruses in vitro (Table 2). However, AF4H1K1 was protective against both H3 and H7 IAVs in vivo (Fig. 5). The successful isolation of AF4H1K1 suggests that NGS-based method is a feasible alternative approach for bnMAb’s screening. This NGS-based method is different from the random mixing and pairing in the phage display, as the heavy chains and light chains are selected from the top of the list based on the frequency and length of CDR3 sequence. These selected heavy and light chains are most probably induced by the H7N9 IAV infection.

It was demonstrated that AF4H1K1 could bind all subtypes of soluble HA proteins from group 2 IAVs, such as H3, H4, H14, H7, H10, and H15 (Supplementary Table S3). Moreover, AF4H1K1 also showed the binding ability to HA proteins expressed on group 2 IAV-infected cell surface (Supplementary Fig. S1a, b). Although AF4H1K1 cannot efficiently neutralize H7 subtype viruses in vitro, it provides good protection against H7N9 IAV in vivo (Fig. 5), indicating it may function through the mechanism of antibody-dependent cellular cytotoxicity (ADCC) or complement-dependent cytotoxicity. ADCC-mediated protection could also be found in other reported bnMAbs targeting the highly conserved HA stem such as FI6, CR9114, 6F12, 2G02, 2B06, and 1F02^16,26,30^.

To understand why only light chain K1 could match with heavy chain H1 to gain the HA-binding and neutralizing activity, we solved the Fab structure of another non-binding pair, AF4H1L1. It was shown that the LCDR3 makes a great contribution to the conformational change of HCDR3, which in turn determines the MAb’s binding and neutralizing activity. This result indicated that light chain has a very important role in MAb’s activity by modulating the conformation of heavy chain, even though the light chain makes little direct interaction with the antigen. Besides, we introduced K1 CDR3 residues into L1 to generate one mutant AF4H1/L1CDR3m. As yet, this mutant did not show any HA-binding or IAV-neutralizing activities. Moreover, AF4H1/FI6v3L, a hybrid MAb with heavy chain from AF4H1K1 and light chain from FI6v3, extends the neutralizing breadth for both H3- and H7-clade IAVs in vitro. The FI6v3 light chain may increase the binding and neutralizing spectrum of AF4H1/FI6v3L by intruding its long LCDR1 loop into the neighboring HA monomer. Based on the binding and neutralization results of AF4H1L1, AF4H1/L1CDR3m, AF4H1K1, and AF4H1/FI6v3L, as well as the structure analysis, we can summarize that the whole light chain has a pivotal role in determining antigen binding and virus neutralization. However, LCDR3 alone is not enough to build up the strength to change IgG behavior.

Previous studies demonstrated that a certain heavy chain paired with a range of light chains could result in the changes of binding affinity to antigens^23,31–33^ and even changed the neutralization activity^28,34^. It is also shown that swapping of heavy chain and light chain of anti-group 1 and group 2 IAV bnMAbs can result in the abolishment of the antigen-binding activity^23^. However, the structural basis has not been characterized in that study. Here, for the first time, our study reveals that light chain could contribute to the binding and neutralizing spectrum by modulating the conformation of paired heavy chain.

Our findings have enhanced the understanding of light-chain contribution to the protection profile of bnMAb against IAVs at molecular level and have opened opportunities for the engineering of more powerful bnMAbs by targeting the light chain.

## Materials and Methods

### Cells and viruses

Human embryonic kidney 293T cells, Madin–Darby canine kidney cells (MDCK), MDCK-SIAT1 cell (MDCK cells with the overexpressing of α-2,6-sialyltransferase, kindly provided by Professor Yuelong Shu), and baby hamster kidney BHK21 cells were cultured in Dulbecco’s modified Eagle’s medium (DMEM) (Invitrogen) supplemented with 10% fetal calf serum (FCS) (Hyclone). IAVs tested in this study include H1N1 A/California/04/2009 (CA/09), H2N3 A/environment/Guangdong/2/2009 (GD/09), H3N2 A/Jiangxi/262/2005 (JX/05), H3N2 A/Beijing-Huairou/11787/2014 (BJ/14), H3 reassortant virus rAC/68 (*HA* from H3N2 A/Aichi/2/1968 and the remaining seven gene segments from H1N1 A/Puerto Rico/8/1934 (PR8)), H4 reassortant virus rCZ/56 (*HA* and *NA* from H4N6 A/duck/Czech/1956, the rest segments from PR8), H5N1 A/bar-headed goose/Qinghai/1/2005 (QH/05), H7N9 A/Anhui/1/2013 (AH/13), H7N9 A/Guangdong/Th005/2017, H9N2 A/chicken/Beijing/2/1997 (BJ/97) H10N8 A/Jiangxi-Donghu/346/2013 (JX/13), H14N5 A/mallard duck/Astrakham/263/1982 (AS/82), and H15N8 A/duck/Australia/341/1983 (AU/83). IAVs stocks were titrated with MDCK or MDCK-SIAT1 cells in 96-well plates as described in the World Health Organization protocol in the presence or absence of TPCK-trypsin (2.5 μg/ml). Fifty percent tissue culture infective dose (TCID_50_) of virus stock was calculated by the Reed–Muench method.

### Recombinant HA proteins

Different baculovirus-derived HA proteins including H1-CA/09, H3-AC/68, H3-JX/05, H3-TX/12 (H3N2 A/Texas/50/2012), H3-BJ/14, H3-HK7276/14 (H3N2 A/Hong Kong/7276/2014), H3-HK7278/14 (H3N2 A/Hong Kong/7278/2014), H4-CZ/56, H5-BG/05, H7-AH/13, H10-JX/13, H14 (H14N6 A/long-tailed duck/Wisconsin/10OS3912/2010), and H15-AU/83 were used to detect the interactions with HuMAbs. The construction of recombinant HA expression plasmids was based on the assay previously described^35,36^. Briefly, the ectodomain of HAs was inserted into the baculovirus transfer vector pFastBac1 (Invitrogen), with a GP67 signal peptide at the N terminus, a thrombin cleavage site, a trimerizing sequence, and a 6xHis tag at the C terminus. Recombinant baculoviruses expressing HAs were amplified in Sf9 insect cells (Invitrogen) and the secreted HAs proteins were collected from Hi5 insect cells (Invitrogen). HA protein purification followed a procedure of Ni-NTA affinity chromatography (GE Healthcare), dialysis, ion-exchange with MonoQ^TM^ 4.6/100 PE (GE Healthcare), Superdex 200 10/300 gel filtration chromatography (GE Healthcare), trimerizing sequence, and His tag removed with thrombin. After removing the trimerizing tag, monomeric HAs were further purified by gel filtration chromatography. Purified HA proteins were stored in the buffer of 20 mM Tris/HCl (pH 8.0), 50 mM NaCl for the antibody–HA complex crystal screening. To perform the SPR experiments, posphate-buffered saline (PBS) containing 0.005% Tween 20 (PBST) would be used as the final buffer.

### Clinical specimens

Following the laboratory confirmation of H7N9 IAV infection for one 21-year-old female, who was administrated in the intensive care unit, fresh blood sample was collected from this patient. Isolated PBMCs from the blood were used for the cDNA amplification of VH and VL. Written informed consent from the guardian was obtained before sample collection. This study was approved by Suzhou Centre for Disease Control and Prevention, China.

### Amplification of VH and VL repertoires

PBMCs were lysed with Trizol (Thermo Scientific) and total RNA was extracted according to the user manual. The quality and quantity of the extracted RNA were assessed using agarose gel electrophoresis and Agilent BioAnalyzer 2100. The first-strand cDNA was synthesized with oligo dT primer by Maxima H Minus First Strand cDNA Synthesis Kit (Thermo Scientific) according to manufacturer’s protocol. Three separate PCR reactions for amplification of VH and VL regions were performed using multiple PCR degenerate primers. Forward VH primers and all reverse primers were cited from Jiang’s research^37^ and forward and reverse κ- and λ-VL primers were designed in the FR1 regions and first 100 nucleotides of constant regions of human κ- and λ genes respectively, based on a total of 161 VL sequences downloaded from IMGT/GENE-DB. The primer sequences are listed in Supplementary Table S5. The PCR products were purified by QIAquick Gel Extraction Kit (Qiagen).

### NGS of VH and VL repertoires

VH and VL libraries were prepared using NEBNext® Ultra™ DNA Library Prep Kit for Illumina (NEB) following the manufacturer’s instruction. After library purification with AgenecourtAMPure XP reagent (Beckman Coulter) and quality control on a Bioanalyzer 2100 High Sensitivity DNA chip (Agilent), NGS libraries were sequenced on the Illumina MiSeq using 2 × 250 configuration.

### Bioinformatics analysis

Raw 2 × 250 MiSeq data were cleaned by Trimmomatic-0.30 (phred33, LEADING: 20, TRAILING: 20, SLIDINGWINDOW: 20:20, MINLEN: 200). The cleaned read1 and read2 sequences were combined by Pandaseq. Framework region (FR) and CDR were identified by IgBlast using IMGT as the reference database. CDR3 was determined by motif WG*G or *GQG (heavy chain) and FG*GT (light chain) at the C terminus of CDR3. After IgBlast, the frequency of CDR3 amino acid sequences was calculated and ranked. The V genes containing the top three ranking of VH CDR3, κ- chain CDR3, and λ-chain CDR3 were selected. The V gene germlines and somatic mutations were identified by IMGT/V-QUEST.

### Construction of antibody genes

Germline leader sequences and germline FR1 sequences were used to obtain the full-length V genes. KOZAK sequence was added in front of the leader sequence. Then the full-length V genes with KOZAK sequence were synthesized (GENEWIZ). CH1 and Fc fragment were added to VH gene and light chain conserved region was added to VL gene by fusion PCR; Fusion PCR primers are listed in Supplementary Table S5. The full-length V genes were cloned to expression vector pCAGGS (kindly provided by Professor Yoshihiro Kawaoka) for expression in mammalian cell. To replace L1 CDR3 residues with the one from K1, site-directed mutagenesis was carried out with specific primers by overlapping PCR extension. Following the steps of restriction digestion and DNA ligation, the mutant gene *L1CDR3m* was cloned to pCAGGS.

### Recombinant IgG and Fab production in mammalian cells

Different pairs of heavy (H) chain and light (L) constructs were co-transfected in 293T cells using polyethylenimine (Alfa Aesar). For the IgG expression the plasmids ratio of H to L chain was 2:1 and for the Fab production the ratio was 1:1. Culture supernatants were collected 4–5 days after transfection and then filtered through a 0.45 μm filter. Antibodies were first purified by protein A affinity chromatography (GE Healthcare) or Ni-NTA affinity chromatography. Then the peak fractions were collected and further purified by gel filtration using a Superdex 200 10/300 GL column. The fractions containing IgG or Fab proteins were pooled, concentrated, and quickly frozen at − 80 °C.

### AF4H1K1 scFv production in *Escherichia coli*

The condon-optimized VH and VL genes of AF4H1K1 were synthesized and cloned into pET21a, a vector for protein expression in *E. coli* by GENEWIZ, China. One linker GGGGSGGGGSGGGGSGGGGS was inserted between VL and VH genes. AF4H1K1 scFv (VL-linker-VH) was expressed in *E. coli* strain BL21 (DE3) as inclusion bodies and then was refolded in vitro as previously described^38^. Briefly, aliquots of inclusion body were diluted dropwise into a stirring refolding system (100 mM Tris, 400 mM L-Arg HCl, 2 mM EDTA, 5 mM reduced glutathione, 1 mM oxidized glutathione, pH 8.0) for overnight at 4 °C. Subsequently, the refolding system was concentrated with a 10 kDa cutoff membrane and then buffer-exchanged into 20 mM Tris-HCl (pH 8.0), 150 mM NaCl. The refolded proteins were further purified by size-exclusion chromatography using a HiLoad 16/60 Superdex 200 pg column (GE Healthcare).

### Enzyme-linked immunosorbent assay

Corning 96-well plates (Sigma-Aldrich, 9018) were coated with purified HA proteins at 200 ng/well in the coating buffer (0.1 M NaHCO_3_, pH 8.6) overnight at 4 °C. Then three following steps, including blocking with 5% bovine serum albumin (w/v), incubation with serial diluted IgGs, and incubation with horseradish peroxidase-conjugated goat-anti-human IgG were carried out. At every step, a volume of 100 μl was added to each well and incubated for 1 h at room temperature (RT). Between incubations, plates were washed three times with PBST buffer. After three incubations, enzymatic activity was measured with substrate 3, 3′, 5′, 5′-tetramethylbenzidine (CWBio) and then stopped by adding 2 M H_2_SO_4_. Absorbance at 450 nm was measured to quantify the binding activity. Binding curves were generated using GraphPad Prism 5 software.

### Gel filtration survival detection

Recombinant HA proteins were mixed with purified Fab fragments at a molar ratio of 1:3 and incubated at 4 °C overnight for complex formation in 20 mM Tris/HCl (pH 8.0), 50 mM NaCl buffer. The samples from individual HA, Fab, and their mixtures were then loaded onto a Superdex 200 10/300 GL column (GE Healthcare), respectively. The chromatographs were recorded and overlaid. The pooled proteins from each peak fraction were analyzed on 12% SDS-PAGE gel and stained with Coomassie blue.

### SPR analysis

The binding kinetics of MAbs with different HAs were evaluated by SPR analysis on Biacore 3000 (GE Healthcare). Purified AF4H1K1 Fab proteins were immobilized directly onto a CM5 sensor chip with standard amine coupling to a surface density about 500 response units. Different concentrations of monomeric HA proteins in PBST buffer were injected sequentially to the modified sensor chip at 30 μl/ml for 2 min, followed by a 6 min dissociation phase to identify binding affinities. *K*_D_ values were calculated based on the binding data using the BIAcore evaluation software.

### Binding of AF4H1K1 to membrane expressed HAs from group 1 and group 2 IAVs

MDCK or MDCK-SIAT1 cells cultured on coverslips were infected with 100 TCID_50_ of IAVs. At 8 h after infection, cells were washed three times with PBS and then incubated with 40 μg/ml purified AF4H1K1 IgG at 37 °C for 1 h. Next, cells were rinsed three times with PBS and fixed with 4% formaldehyde for 15 min at RT. Following three more washes with PBS, the cells were permeabilized with 0.2% Triton X-100 for 5 min. After removing the Triton solution, three rinses with PBS were done and goat serum was added to the coverslips for 1 h at RT to block the nonspecific adsorption. Cells were subsequently incubated with mouse anti-NP MAb for 1 h at RT. Cells were then rinsed again with PBST as above and fluorescein isothiocyanate-labeled goat-anti-human IgG, Alexa Fluor 488-labeled goat-anti-mouse IgG, and 4′,6′-diamidino-2-phenylindole hydrochloride were then added to cells and incubated for 1 h at RT. After three rinses with PBST, binding of AF4H1K1 to influenza virus-infected cells was analyzed using confocal microscope (Leica TCS SP5 II).

### Virus neutralization

Microneutralization test was carried out in MDCK cells or MDCK-SIAT1 cells based on the assays previously described^39^. Briefly, 50 μl 200 TCID_50_ of virus inoculum was mixed with equal volume of a twofold dilution series of antibody. Triplicates were set up for each antibody dilution. Following incubation for 1 h at 37 °C in 5% CO_2_, 100 μl virus–antibody mixture was inoculated into MDCK or MDCK-SIAT1 cells in each well with a 96-well culture plate. After 3–4 days incubation at 37 °C in 5% CO_2_, cytopathic effect in each well was observed and the hemagglutination activity of the cell cultures was also detected. Nonlinear regression was applied for neutralization curve fit using GraphPad Prism version 5.0 and the IC_50_ were determined accordingly.

### Evaluation of prophylactic and therapeutic efficacy in mouse

All experiments with mice were approved by the Ethics Committee for animal experimentation of the Institute of Microbiology, Chinese Academy of Sciences. In the prophylactic experiments with H3 IAV infection, groups of fifteen 6-week-old female BABL/c mice were administrated intravenously with purified AF4H1K1 IgG at different concentrations, such as 15, 10, 3, and 1 mg/kg. Twenty-four hours later, the mice were anesthetized with solid CO_2_ and challenged intranasally with 100 MID_50_ H3-rAC/68 reassortant virus. In the therapeutic experiments with H3 IAV infection, antibody with a dose of 15 mg/kg was administrated intravenously at 4, 24, and 48 h respectively, after virus challenge. After virus infection in both prophylactic and therapeutic evaluation tests, five mice were killed at 3 and 5 dpi, respectively, to collect lung tissues. The lung tissues were homogenized in DMEM medium supplemented with Penicillin–Streptomycin to achieve 10% w/v suspension. Supernatants from centrifuged homogenized lung tissues were titrated on MDCK cells and virus titers were determined. In the prophylactic and therapeutic experiments with H7N9 IAV, groups of five 6-week-old female BABL/c mice were administrated with various MAbs intravenously through the tail veil at 24 h before and after virus challenge, respectively. The body weight and survival were monitored on daily basis until 14 dpi. Animal experiments with H7N9 HPAIV were conducted at Biosafety level 3 laboratory.

### Fusion inhibition in virus-infected cells

BHK21 cells seeded in 24-well plate with coverslipes were infected with H3-rAC/68 at a multiplicity of infection of 5. After 12 h post infection, virus-infected cells were incubated with 2.5 μg/ml TPCK-treated trypsin (Sigma) for 5 min and then DMEM containing 10% FCS was added to inactivate the trypsin. Cell cultures were treated with AF4H1K1 at the concentration of 40 μg/ml for 1 h at 37 °C. Following three rinses of PBS and one wash with pre-warmed low-pH fusion buffer (citric acid–sodium citrate buffer at pH 5.0), virus-infected cells were incubated with low-pH fusion buffer for 5 min at 37 °C. Cells were then returned to the standard culture medium for 1–3 h at 37 °C and fixed with 4% formaldehyde for 20 min. Syncytium formation was photographed using confocal microscope (Leica TCS SP5 II).

### Inhibition of HA0 trypsin-mediated cleavage by AF4H1K1

Those baculovirus-derived HA0 proteins, such as H3 HA0, H4 HA0, H14 HA0, and H15 HA0, were incubated with different amounts of AF4H1K1 for 40 min at 37 °C. TPCK-treated trypsin was then added to each sample to a final concentration of 5 μg/ml and protease digestion was performed at 37 °C for 15 min before being stopped by adding a loading buffer containing SDS and dithiothreitol, and then by boiling them at 95 °C for 5 min. Samples were then loaded on a 12% SDS-PAGE gel.

### Inhibition of mature HA conformation change by AF4H1K1

Baculovirus-derived immature HAs, such as H3 HA0, H4 HA0, H14 HA0, and H15 HA0, were treated with trypsin at a final concentration of 5 μg/ml at 4 °C overnight. Then, the mature HAs were further purified by gel filtration to remove the trypsin. Purified mature HA proteins were incubated with AF4H1K1 IgG at the 1:1 molar ratio for 4 h at 4 °C before being adjusted to pH 5.0 with sodium acetate acid buffer. After incubation for 1 h at 37 °C in pH 5.0, 1 M Tris/HCl buffer (pH 8.0) was added into the HA/AF4H1K1 mixture to bring back the pH to 7.4. Trypsin was then added to the mixture at a final concentration of 20 μg/ml and the mixture was incubated for 4 h at 4 °C. The reaction was terminated by adding a loading buffer containing SDS. Samples were then loaded on a 12% SDS-PAGE gel after being boiled at 95 °C for 5 min.

### AF4H1K1/H3, AF4H1K1/H4, AF4H1K1 scFv, and AF4H1L1 Fab crystallization and structure determination

Recombinant H3 or H4 HA proteins were mixed with purified Fab fragment of AF4H1K1 at a molar ratio of 1:5 and incubated at 4 °C overnight for complex formation in 20 mM Tris/HCl (pH 8.0), 50 mM NaCl buffer. The mixture was then loaded onto a Superdex 200 10/300 GL column to purify the Fab–HA complex from any excess Fab. Peak fractions corresponding to the Fab–HA complex were collected and concentrated for crystallization. AF4H1K1/H3 complex crystals, AF4H1K1/H4 complex crystals, and AF4H1K1 scFv and AF4H1L1 Fab crystals were grown by vapor diffusion in sitting drops. A total of 1.0 μl of Fab–HA complex, scFv, or Fab protein solution at 5 or 10 mg/ml was mixed with an equal volume of reservoir solution. A total of 0.3 μl of AF4H1K1 scFv protein solution at 5 or 10 mg/ml was mixed with the same volume of reservoir. Good quality crystals of AF4H1K/H3 were obtained with the condition of 0.19 mM CYMAL-7, 0.1 M HEPES pH 7.5, 40% v/v ployethylene glycol 400 at 18 °C, and diffracted at 2.9 Å resolution. Crystals of AF4H1K/H4 complex grew at the buffer solution of 0.2 M potassium sodium tartrate tetrahydrate, 0.1 M Bis-Tris pH 6.5, 10% w/v polyethylene glycol 10,000, and diffracted at 3.8 Å resolution. Good quality crystals of AF4H1K1 scFv were obtained with the condition of 1.4 M sodium malonate, 0.1 M Bis-Tris propane pH 7.0 at 18 °C, and diffracted at 1.4 Å resolution. Crystals of AF4H1L1 Fab were obtained with the condition of 0.1 M Bicine pH 9.0, 2% v/v 1,4-Dioxane, 10% w/v PEG20000, and diffracted at 2.1 Å resolution.

Crystals were frozen in the same reservoir solution with 25% glycerol.

The data sets of the AF4H1K1/H3 and AF4H1K1/H4 were collected at the Shanghai Synchrotron Radiation Facility beam line 17U1, whereas the data set of AF4H1K1 scFv and AF4H1L1 Fab were both collected at beam line 19U1. All of the data sets were processed using HKL2000 package. The structures were solved by molecular replacement using Phaser in the CCP4 suit and refined with Phenix program. All data collection and refinement statistics are summarized in Supplementary Table S4.

Crystal data were deposited in PDB with the submission ID: 5Y2L for AF4H1K1/H3 complex, 5Y2M for AF4H1K1/H4 complex, 5Y2K for AF4H1L1 Fab, and 6J9O for AF4H1K1 scFv.

### Statistical analysis

GraphPad Prism version 5.0 (GraphPad Software, Inc.) was used for the statistical analysis in this study.

## Supplementary information

Supplementary Information

Supplementary Table S5

## Supplementary information

**Supplementary Information** accompanies the paper at (10.1038/s41421-019-0086-x).

## References

[1] Johnson NP, Mueller J (2002). Updating the accounts: global mortality of the 1918-1920 “Spanish” influenza pandemic. Bull. Hist. Med..

[2] Reid AH, Taubenberger JK (2003). The origin of the 1918 pandemic influenza virus: a continuing enigma. J. Gen. Virol..

[3] Novel Swine-Origin Influenza A (H1N1) Virus Investigation Team.. (2009). Emergence of a novel swine-origin influenza A (H1N1) virus in humans. N. Engl. J. Med..

[4] Itoh Y (2009). In vitro and in vivo characterization of new swine-origin H1N1 influenza viruses. Nature.

[5] Fukumi H (1959). Summary report on the Asian influenza epidemic in Japan, 1957. Bull. World Health Organ..

[6] Henderson DA, Courtney B, Inglesby TV, Toner E, Nuzzo JB (2009). Public health and medical responses to the 1957-58 influenza pandemic. Biosecur. Bioterror..

[7] Schulman JL, Kilbourne ED (1969). Independent variation in nature of hemagglutinin and neuraminidase antigens of influenza virus: distinctiveness of hemagglutinin antigen of Hong Kong-68 virus. Proc. Natl Acad. Sci. USA.

[8] Shi Y, Wu Y, Zhang W, Qi JX, Gao GF (2014). Enabling the ‘host jump’: structural determinants of receptor-binding specificity in influenza A viruses. Nat. Rev. Microbiol..

[9] Impagliazzo A (2015). A stable trimeric influenza hemagglutinin stem as a broadly protective immunogen. Science.

[10] Krammer F, Pica N, Hai R, Margine I, Palese P (2013). Chimeric hemagglutinin influenza virus vaccine constructs elicit broadly protective stalk-specific antibodies. J. Virol..

[11] Sautto GA, Kirchenbaum GA, Ross TM (2018). Towards a universal influenza vaccine: different approaches for one goal. Virol. J..

[12] Yassine HM (2015). Hemagglutinin-stem nanoparticles generate heterosubtypic influenza protection. Nat. Med..

[13] Sui JH (2009). Structural and functional bases for broad-spectrum neutralization of avian and human influenza A viruses. Nat. Struct. Mol. Biol..

[14] Yoshida R (2009). Cross-protective potential of a novel monoclonal antibody directed against antigenic site B of the hemagglutinin of influenza A viruses. PLoS Pathog..

[15] Ekiert DC (2009). Antibody recognition of a highly conserved influenza virus epitope. Science.

[16] Corti D (2011). A neutralizing antibody selected from plasma cells that binds to group 1 and group 2 influenza A hemagglutinins. Science.

[17] Ekiert DC (2011). A highly conserved neutralizing epitope on group 2 influenza A viruses. Science.

[18] Ohshima N (2011). Naturally occurring antibodies in humans can neutralize a variety of influenza virus strains, including H3, H1, H2, and H5. J. Virol..

[19] Ekiert DC (2012). Cross-neutralization of influenza A viruses mediated by a single antibody loop. Nature.

[20] Wu Y (2015). A potent broad-spectrum protective human monoclonal antibody crosslinking two haemagglutinin monomers of influenza A virus. Nat. Commun..

[21] Tharakaraman K (2015). A broadly neutralizing human monoclonal antibody is effective against H7N9. Proc. Natl Acad. Sci. USA.

[22] Gupta P (2016). Preclinical pharmacokinetics of MHAA4549A, a human monoclonal antibody to influenza A virus, and the prediction of its efficacious clinical dose for the treatment of patients hospitalized with influenza A. MAbs.

[23] Joyce MG (2016). Vaccine-induced antibodies that neutralize group 1 and group 2 influenza A viruses. Cell.

[24] Kallewaard NL (2016). Structure and function analysis of an antibody recognizing all influenza A subtypes. Cell.

[25] Geskey JM, Thomas NJ, Brummel GL (2007). Palivizumab: a review of its use in the protection of high risk infants against respiratory syncytial virus (RSV). Biologics.

[26] Dreyfus C (2012). Highly conserved protective epitopes on influenza B viruses. Science.

[27] Collet TA (1992). A binary plasmid system for shuffling combinatorial antibody libraries. Proc. Natl Acad. Sci. USA.

[28] Huang JH (2016). Identification of a CD4-binding-site antibody to HIV that evolved near-pan neutralization breadth. Immunity.

[29] Zhang F (2017). Human infections with recently-emerging highly pathogenic H7N9 avian influenza virus in China. J. Infect..

[30] DiLillo DJ, Tan GS, Palese P, Ravetch JV (2014). Broadly neutralizing hemagglutinin stalk-specific antibodies require FcγR interactions for protection against influenza virus in vivo. Nat. Med..

[31] Radic MZ, Mascelli MA, Erikson J, Shan H, Weigert M (1991). Ig H and L chain contributions to autoimmune specificities. J. Immunol..

[32] Suarez E (2006). Rearrangement of only one human IGHV gene is sufficient to generate a wide repertoire of antigen specific antibody responses in transgenic mice. Mol. Immunol..

[33] Wiehe K (2014). Antibody light-chain-restricted recognition of the site of immune pressure in the RV144 HIV-1 vaccine trial is phylogenetically conserved. Immunity.

[34] Zhou TQ (2013). Multidonor analysis reveals structural elements, genetic determinants, and maturation pathway for HIV-1 neutralization by VRC01-class antibodies. Immunity.

[35] Zhang W (2010). Crystal structure of the swine-origin A (H1N1)-2009 influenza A virus hemagglutinin (HA) reveals similar antigenicity to that of the 1918 pandemic virus. Protein Cell.

[36] Lu X (2013). Structure and receptor-binding properties of an airborne transmissible avian influenza A virus hemagglutinin H5 (VN1203mut). Protein Cell.

[37] Jiang N (2013). Lineage structure of the human antibody repertoire in response to influenza vaccination. Sci. Transl. Med.

[38] Dai L (2016). Structures of the Zika virus envelope protein and its complex with a flavivirus broadly protective antibody. Cell. Host. Microbe.

[39] Song XH (2010). Serological surveillance of influenza A virus infection in swine populations in Fujian Province, China: no evidence of naturally occurring H5N1 infection in pigs. Zoonoses Public. Health.

