# Supplementary material for: Light chain modulates heavy chain conformation to change protection profile of monoclonal antibodies against influenza A viruses
Source: Cell Discov. 2019 Apr 16;5:21. doi: 10.1038/s41421-019-0086-x (PMC6465249; doi:10.1038/s41421-019-0086-x)
Supplement: Supplementary file 1 — Supplementary Information [file 41421_2019_86_MOESM1_ESM.pdf]

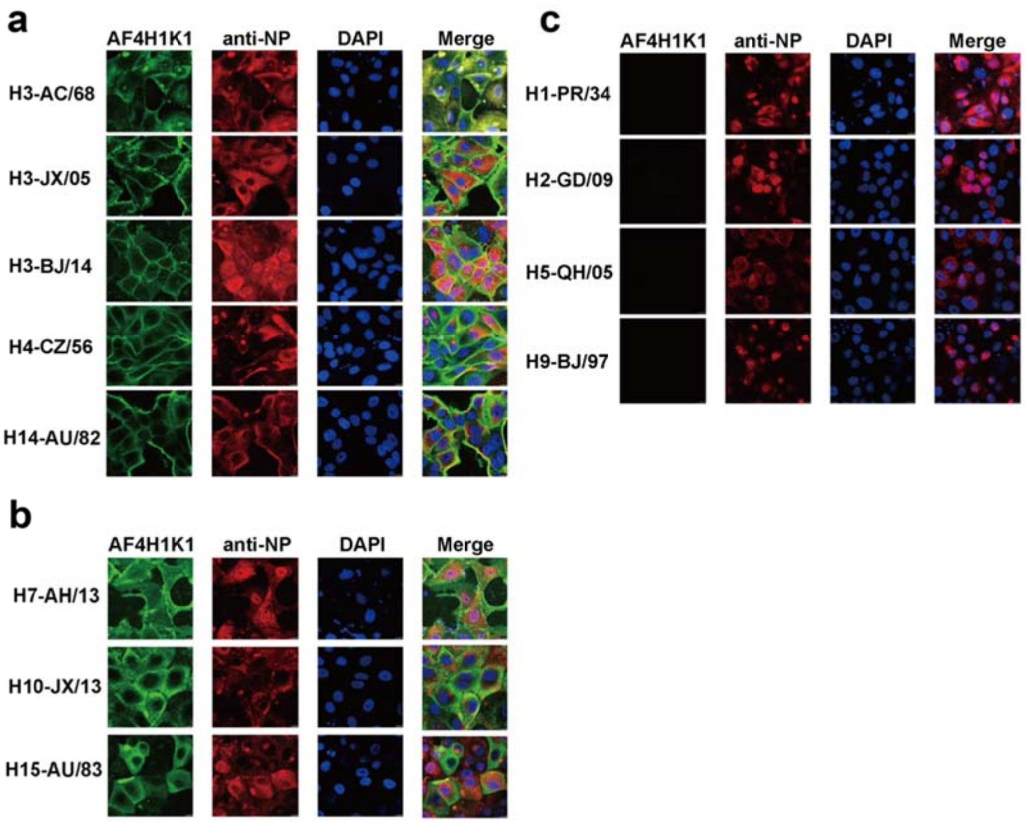

Fig. S1 Binding spectrum of AF4H1K1 to the HAs expressed in divergent IAVs-infected cell surface.

AF4H1K1 recognizes HA proteins from H3-clade of group 2 IAVs **(a)**, H7-clade of group 2 IAVs **(b)**, but not HAs from group 1 IAVs **(c)**, which were detected by Immunofluorescence assay (IFA). IAVs nuclear protein (NP) in virus-infected cells was stained with anti-NP mouse MAb (red) in contrast to the cell nuclear staining (blue). HAs in the virus-infected cells were stained with AF4H1K1 are in green. Bar, 100 μm.

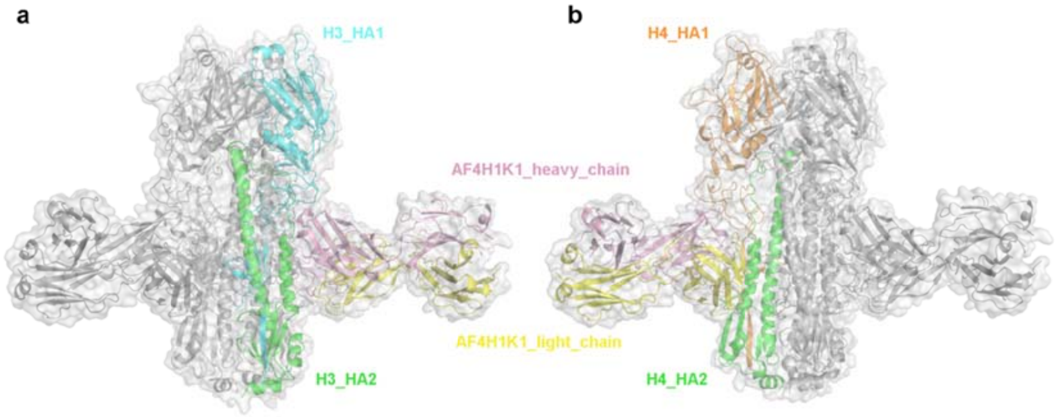

Fig. S2 Overall structures of AF4H1K1 Fab in complex with H3/H4 HA molecules.

The HA trimers bound to Fab molecules are shown as ribbon and surface models. Only one HA protomer bound to a Fab fragment is highlighted in different colors and the remaining portions are colored in grey. The heavy and light chains of Fab fragment are colored in pink and yellow respectively. The HA1 subunits in H3 **(a)** and H4 **(b)** are shown in cyan and orange respectively, and the HA2 subunits in both structures are colored in green.

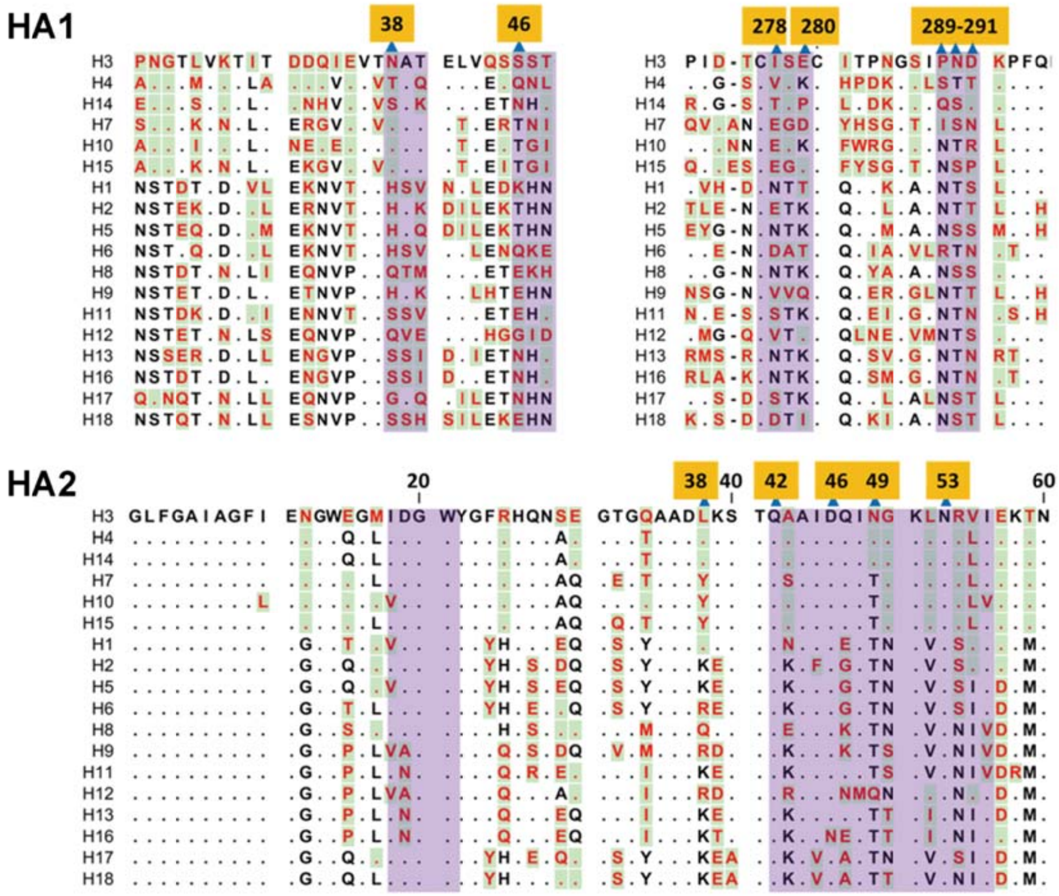

Fig. S3 Amino acid sequence alignment of H1-H18 HA proteins.

Eighteen subtypes of HA protein sequence were aligned using the CLC Sequence Reviewer software. The main regions of HA interacting with AF4H1K1 antibody are covered with purple shadows.

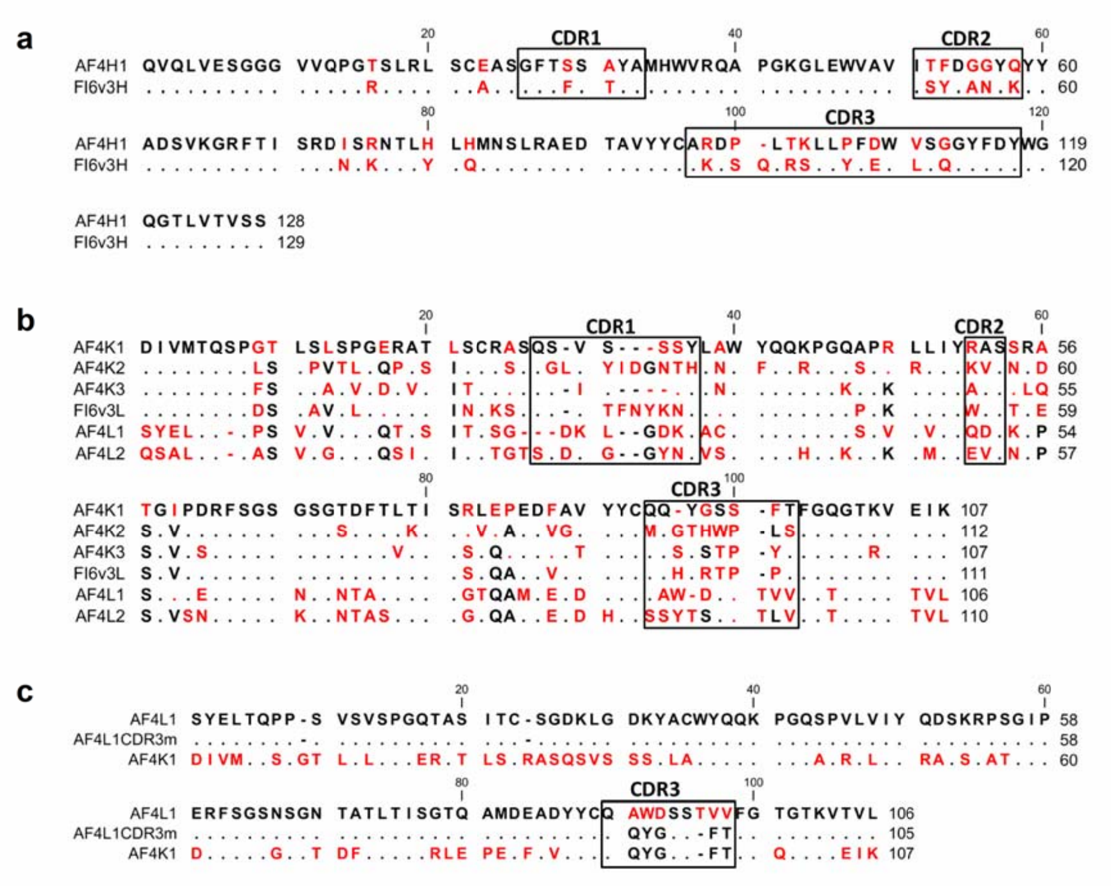

Fig. S4 Sequence alignment of different heavy and light chain variable regions.

(**a**) Amino acid sequence alignment between AF4H1K1 and FI6v3 VH regions using the CLC Sequence Reviewer software. The main differences in the HCDR regions are highlighted with rectangles. (**b**) Amino acid alignment among different light chain variable regions using CLC Sequence Reviewer software. Different LCDR regions are covered with rectangles, FI6v3 light chain is represented by FI6v3L in this alignment. **(c)** Amino acid alignment among L1, L1CDR3m and K1 variable regions using CLC Sequence Reviewer software. LCDR3 regions are highlighted with rectangle.

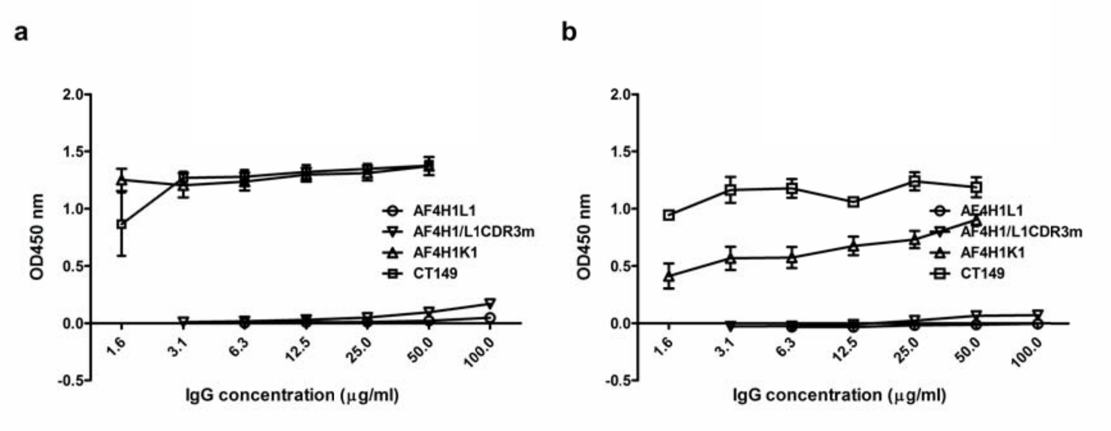

Fig. S5 Binding ability of AF4H1/L1CDR3m to H3 and H7 HA proteins.

Binding ability of mutant AF4H1/L1CDR3m with H3HA (H3-AC/68) **(a)** or H7HA (H7-AH/13) **(b)** was determined by ELISA assay. In this assay, CT149, a positive control (rectangle), AF4H1K1 (triangle) and wide type AF4H1L1 (circle) were tested in paralleled with AF4H1/L1CDR3m (inverted triangle). The data were summarized with three repeated experiment results, and were graphed using GraphPad Prism 5 software.

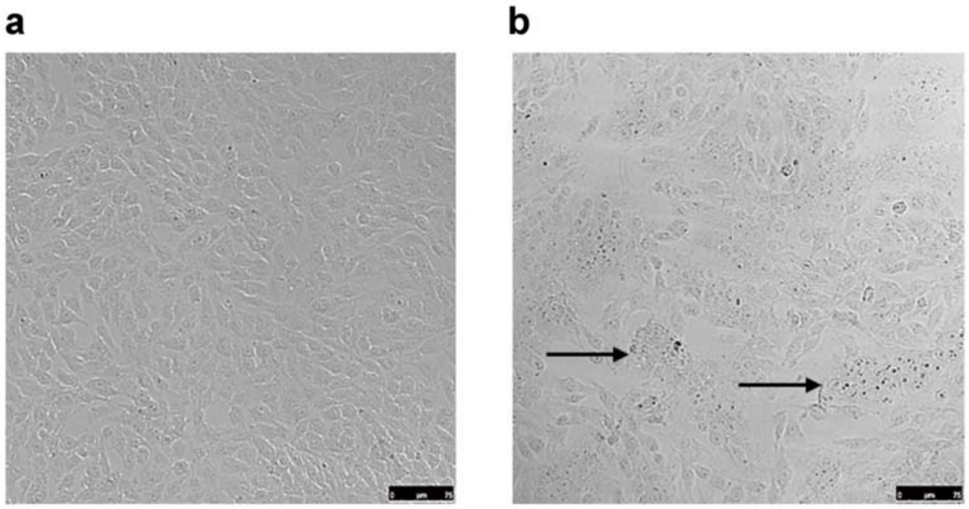

Fig. S6 AF4H1K1 inhibits syncytia formation.

BHK21 cells infected with H3-JX/05 were briefly exposed to pH5.0 at 37°C in the presence of AF4H1K1 **(a)** or the negative control IgG **(b)** at a concentration of 40 μg/ml. Polykaryon formation could not be inhibited in the negative IgG treated cells (arrows). Bar, 100 μm.

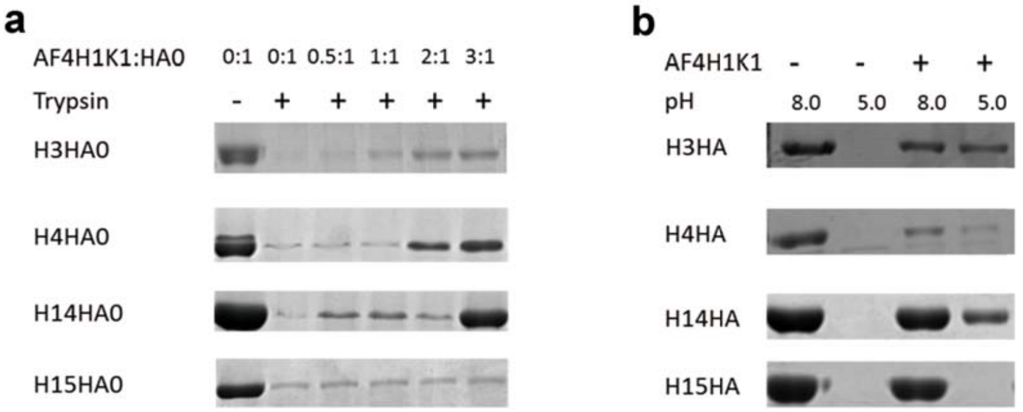

Fig. S7 AF4H1K1 inhibits the HA0 cleavage and mature HA conformational change.

(a) AF4H1K1 inhibits the HA0 cleavage. Recombinant HA0s from H3, H4, H14 and H15 were incubated with AF4H1K1 in different molar ratios. The AF4H1K1 and HA0 mixture was exposed to TPCK-treated trypsin for 15 min at 37°C, and then was loaded on SDS-PAGE gel. **(b)** AF4H1K1 inhibits the HA conformational change. Mature HAs, including H3, H4, H14 and H15 were incubated with AFH1K1 before the buffer pH dropped to 5.0, and then the low-pH buffer-treated HAs were exposed to TPCK-treated trypsin.

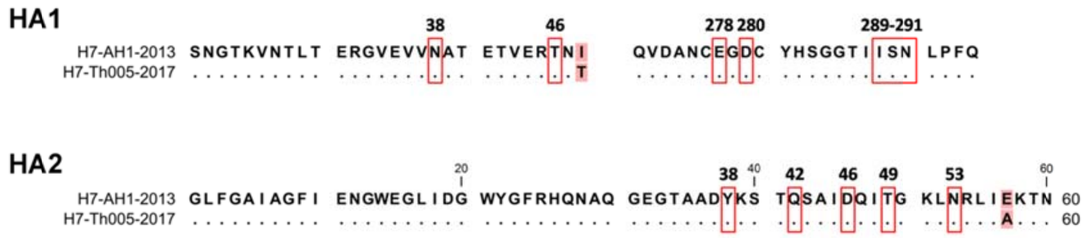

Fig. S8 Amino acid alignment of HA proteins respectively from 2013 H7N9 LPAIV (A/Anhui/1/2013, H7-AH1-2013) and 2017 H7N9 HPAIV (A/Guangdong/Th005/1/2017, H7-Th005-2017). Sequence alignment was performed using CLC Sequence Reviewer software. The main regions interacting with AF4H1K1 are covered with red triangles.

Table S1. Analysis on CDR3 of variable heavy and light chains from an H7N9 infected patient

Variable heavy chain			Variable kappa light chain			Variable lambda light chain		
**CDR3 sequence**	**Number**	**Percentage (%)**	**CDR3 sequence**	**Number**	**Percentage (%)**	**CDR3 sequence**	**Number**	**Percentage (%)**
ARDPLTKLLPFDWVSGGYFDY*	14445	2.8	QQYGSSFT	39013	12.2	QAWDSSTVV	116662	23.0
AHFGQWLVYFDY	13263	2.6	MQGTHWPLS	8104	2.5	QT	41393	8.2
ARDVYDFWSEPFNYDFCYMDV	9719	1.9	QQSYSTPYT	7331	2.3	SSYTSSSTLV	22278	4.4
DVYDFWSEPFNYDYCYMDV	3157	0.6	QQSYNSPPT	7174	2.2	SSYTSSSTVV	13062	2.6
GDVDYDYWSDYEGRRFDP	4687	0.9	QQYNNWPPYT	6325	2.0	QAWDSSTAV	11808	2.3
DKPYLIVGAVIDY	4545	0.9	QQRSNWPPIT	6150	1.9	QAWDSSIVV	11706	2.3
ITDYYGSGSYLGFDY	3919	0.8	MQGTHWPPT	6052	1.9	LLSYSGARPLVV	6509	1.3
GGIVATTEDDAFDI	3177	0.6	CQQYSDWPPYT	5449	1.7	QAWDSSNVV	6488	1.3
DRGHILTGYLMDY	3025	0.6	MQGTHWPLT	4891	1.5	QAWDSSTGV	5215	1.0
GSYYDFWSGYSDYYMDV	3004	0.6	QQYGSSPPLT	4413	1.4	CSYAGRYTWV	4710	0.9
EYYYDSSGYYYLNY	2780	0.5	QQRTYWPPVT	3736	1.2	QVWDSSSDHRYV	4647	0.9
GERGVDYDFWNDISADYYMDV	2555	0.5	QQYGSSPPYT	3365	1.1	CSFTTSDTLI	4495	0.9
DLDSGYDRYYYYGMDV	2552	0.5	QQYNNWPET	3260	1.0	SAWDSSLSAWV	4425	0.9
DLHDYSNYGYYYMDV	2432	0.5	MQGTHWPYT	3232	1.0	SSYTSSTTPVV	3569	0.7
DRRDILSGSYVDY	2396	0.5	QQYNNWPRT	2935	0.9	CSYTSSSTLV	3470	0.7
DHTPYSSGYYYMDV	2380	0.5	QQYSDWPPYT	2830	0.9	CSFTSSDTLI	3048	0.6
GVDDFWSNYKTFDY	2375	0.5	QQRSNWPLT	2679	0.8	MIWHSSAYV	2764	0.5
**Grand total**	**508261**	**100.0**	**Grand total**	**508261**	**100.0**	**Grand total**	**508261**	**100.0**

*VH and VL CDR3 sequences selected for further study are labeled with green background.

Table S2. Expression, gel filtration survival with HAs, and IAVs neutralization activity for different pairs of antibodies from donor AF4.

IgG pairs	Expression in 293T cell	Gel filtration survival with recombinant HAs		Microneutralization with different subtypes of IAVs *in vitro*	
		H3 HA	H7 HA	H3N2	H7N9
**AF4H1K1**	+	+	+	+	-
**AF4H1K2**	-	ND*	ND	ND	ND
**AF4H1K3**	+	-	-	-	-
**AF4H1L1**	+	-	-	-	-
**AF4H1/L1 CDR3m**	+	ND	ND	-	-
**AF4H1L2**	+	-	-	-	-
**AF4H2K1**	+	ND	ND	-	-
**AF4H2K2**	+	ND	ND	-	-
**AF4H2K3**	+	ND	ND	-	-
**AF4H2L1**	+	ND	ND	-	-
**AF4H2L2**	+	ND	ND	-	-
**AF4H3K1**	+	ND	ND	-	-
**AF4H3K2**	+	ND	ND	-	-
**AF4H3K3**	+	ND	ND	-	-
**AF4H3L1**	+	ND	ND	-	-
**AF4H3L2**	+	ND	ND	-	-

*ND means not done

Table S3. SPR analysis of binding affinity between different Fab molecules of IgG pairs and various monomeric recombinant HAs.

Clades	Subtypes	HA donor virus	K_D values (M)		
			**AF4H1L1**	**AF4H1K1**	**AF4H1/FI6v3L**
H3	H3N2	A/Aichi/2/1968	-*	7.9E-10	7.64E-11
	H3N2	A/Jiangxi/262/2005	ND[#]	4.0E-12	ND
	H3N2	A/Texas/50/2012	ND	4.9E-08	ND
	H3N2	A/Beijing-Huairou/11787/2014	ND	3.1E-11	ND
	H3N2	A/Hong Kong/7276/2014	ND	2.4E-08	ND
	H4N6	A/duck/Czech/1956	-	9.0E-09	1.93E-9
	H14N6	A/long-tailed duck/Wisconsin/10OS3912/2010	-	1.6E-08	1.59E-8
H7	H7N9	A/Anhui/1/2013	-	1.4E-08	1.12E-8
	H10N8	A/Jiangxi-Donghu/346/2013	-	2.1E-08	3.77E-8
	H15N8	A/duck/Australia/341/1983	-	1.8E-06	5.18E-7

*AF4H1L1has no binding activity to these HAs.

[#] means not done.

Table S4. Data collection and refinement statistics for the AF4H1K1/H3 complex, AF4H1K1/H4 complex, AF4H1K1 scFv and AF4H1L1 Fab.

	**AF4H1K1/H3**	**AF4H1K1/H4**	**AF4H1K1 scFv**	**AF4H1L1 Fab**
**Data collection**				
Space group	R32	P21	P212121	P21
Cell dimensions				
a, *b*, *c* (Å)	157.48, 147.48, 355.34	114.18, 140.94, 138.10	36.75, 56.80, 102.58	107.27, 90.12, 111.40
α, β, γ (^{o})	90.00, 90.00, 120.00	90.00, 95.31, 90.00	90.00, 90.00, 90.00	90.00, 102.38, 90.00
Resolution(Å)*	50.00-2.90 (3.00-2.90)	50.00-3.80 (3.94-3.80)	50.00-1.40(1.45-1.40)	50-2.10 (2.80-2.10)
Unique reflections	37831 (3739)	43204 (4301)	42860(3818)	118899 (11093)
R_{merge}	0.097 (0.702)	0.255 (0.841)	0.076(0.797)	0.058 (0.343)
R_{pim}	0.028 (0.213)	0.146 (0.485)	0.020(0.243)	0.026 (0.182)
$I/\sigma I$	23.0 (2.5)	6.0 (1.9)	39.3(3.8)	25.1 (4.5)
Completeness (%)	99.9 (100.0)	99.8 (99.9)	98.7(97.5)	98.0 (92.3)
Redundancy	10.7 (10.7)	4.0 (4.0)	14.3(11.1)	6.0 (4.5)
Wilson B factors	69	68	10.4	39
**Refinement**				
Resolution (Å)	47.33-2.90	49.20-3.80	34.59-1.40	41.63-2.10
No. reflections	37724	43072	42545	118836
R_{work} / R_{free}	0.212/0.244	0.236/0.277	0.1764/0.1917	0.220/0.259
No. atoms				
Protein	7189	18315	1857	12904
Ligand/ion	0	0	0	0
Water	0	0	291	566
B-factors				
Protein	86	98	15.3	59
Ligand/ion				
Water			27.2	54.9
R.M.S. deviations				
Bond lengths (Å)	0.008	0.004	0.003	0.005

Bond angles (^{o})	1.164	0.758	0.673	1.026
Ramchandran Statistics (%)				
Favored	92.16	94.23	97.86	96.09
Allowed	7.51	5.04	2.14	3.14
Disallowed	0.33	0.73	0	0.77

*Values in parentheses are for highest-resolution shell.
